# Evidence that informs feeding practices in very low birthweight and very preterm infants in sub-Saharan Africa: an overview of systematic reviews

**DOI:** 10.1136/bmjpo-2020-000724

**Published:** 2020-08-11

**Authors:** Abimbola Akindolire, Alison Talbert, Ian Sinha, Nicholas Embleton, Stephen Allen, on behalf of the Neonatal Nutrition Network (NeoNuNet), Olusegun Akinyinka

**Affiliations:** 1 College of Medicine, University of Ibadan, Ibadan, Oyo, Nigeria; 2 Clinical Research, KEMRI-Wellcome Trust Research Programme, Kilifi, Kenya; 3 Respiratory Medicine, Alder Hey Children’s NHS Foundation Trust, Liverpool, UK; 4 Neonatal medicine, Newcastle University, Newcastle upon Tyne, Tyne and Wear, UK; 5 Paediatrics, Liverpool School of Tropical Medicine, Liverpool, UK

**Keywords:** neonatology

## Abstract

**Background:**

Optimal feeding of very low birthweight (VLBW <1500 g)/very preterm (gestation <32 weeks) infants in resource-limited settings in sub-Saharan Africa (sSA) is critical to reducing high mortality and poor outcomes.

**Objective:**

To review evidence on feeding of VLBW/very preterm infants relevant to sSA.

**Methods:**

We searched the Cochrane Database of Systematic Reviews, Embase, PubMed and Cumulative Index to Nursing and Allied Health Literature (CINAHL) from inception to July 2019 to identify reviews of randomised and quasi-randomised controlled trials of feeding VLBW/very preterm infants. We focused on interventions that are readily available in sSA. Primary outcomes were weight gain during hospital stay and time to achieve full enteral feeds (120 mL/kg/day). Secondary outcomes were growth, common morbidities, mortality, duration of hospital stay and cognitive development. Quality of evidence (QOE) was assessed using the Measurement Tool to Assess Systematic Reviews (AMSTAR2).

**Results:**

Eight systematic reviews were included. Higher feed volume of day 1 (80 mL/kg) reduced late-onset sepsis and time to full enteral feeds, and higher feed volume (up to 300 mL/kg/day) improved weight gain without adverse events (QOE: low–moderate). Rapid advancement of feeds (30–40 mL/kg/day) was not associated with harm. Breast milk fortification with energy and protein increased growth and with prebiotics increased growth and reduced duration of admission (QOE: low–very low) and did not result in harm. Evidence regarding feeding tube placement and continuous versus bolus feeds was insufficient to draw conclusions. We found no reviews meeting our selection criteria regarding when to start feeds, use of preterm formula, cup-and-spoon feeding or gravity versus push feeds and none of the reviews included trials from low-income countries of sSA.

**Conclusions:**

The evidence base informing feeding of VLBW/very preterm babies in resource-limited settings in sSA is extremely limited. Pragmatic studies are needed to generate evidence to guide management and improve outcomes for these highly vulnerable infants.

**PROSPERO registration number:**

CRD42019140204.

**What is known about the subject?**

- Although survival is improving, mortality and outcomes for very low birthweight (VLBW)/very preterm infants in resource-limited settings in sub-Saharan Africa (sSA) remain poor.
- Optimising feeding is critical to improving survival, healthy growth and development and reducing morbidity.
- International and national protocols inform the feeding of VLBW/very preterm infants but evidence relevant to resource-limited settings is limited.

**What this study adds?**

- No trials of pragmatic feeding interventions have been conducted in resource-limited settings in sSA.
- Higher feed volumes, rapid advancement of feeds and fortification of breast milk may be beneficial in achieving full feeds, improving growth and reducing morbidity without adverse effects.
- Trials of pragmatic feeding protocols in resource-limited settings are needed urgently to better inform clinical practice.

## Background

Worldwide about 15 million babies are born preterm annually and it is estimated that 28% of these births occur in sub-Saharan Africa (sSA).^1^ An increasing number of very low birthweight (VLBW; BW <1500 g) babies are surviving till discharge in low/middle-income countries (LMICs).^2^ Optimal nutrition is key for survival, prevention of adverse events such as sepsis and reducing the length of the hospital stay.^3^ In addition, there is strong evidence that early nutrition impacts on multiorgan developmental patterning, immune, cardiac and respiratory function and longer term cognitive outcome as well as a range of chronic conditions such as diabetes and cardiovascular disease in adulthood which place a substantial burden on healthcare systems.^3–7^

Most very preterm infants (gestation <32 weeks) have yet to develop coordinated oromotor skills of sucking, swallowing and breathing that allow safe breast feeding and, therefore, other methods of feeding are necessary. Uncertainty about gestational age due to a lack of reliable first trimester ultrasound scan is common in LMICs and birth weight is often used to identify infants who are preterm, growth retarded or both.^8^ In most neonatal units in LMICs, parenteral nutrition is not available.^9^ Trials on enteral feeding interventions have been done mainly in high-income and middle-income countries. The generalisability of their findings to feeding VLBW infants in most of sSA is difficult to assess due to multiple differences in maternal, pregnancy, delivery, neonatal and environmental factors and also the levels of care and clinical monitoring provided in high-income versus LMICs.^10–12^

Although internationally agreed guidelines on optimal feeding of low birthweight infants in LMICs, including some recommendations for VLBW infants, were published by the WHO in 2011, these lacked a strong evidence base especially for LMIC settings.^9^ This led us to search the literature for evidence from reviews of studies conducted in, or applicable to, sSA. The aim of our study was to review the evidence for pragmatic, low-cost interventions to optimise the feeding of VLBW/very preterm infants in low-resource settings in sSA. We systematically searched for evidence on when to start enteral feeds; how to advance feeds; what to feed when mother’s own milk is insufficient or not available and how to feed.

## Methods and analysis

### Protocol registration

We registered the overview protocol prospectively on the PROSPERO International Prospective Register of Systematic Reviews (http://www.crd.york.ac.uk/PROSPERO/display_record.php?ID=CRD42019140204).

### Patient and public involvement

There was no patient or public involvement in the planning or execution of this overview.

### Search strategy

We searched the Cochrane Database of Systematic Reviews, Embase, PubMed and CINAHL from inception to July 2019 for systematic reviews and meta-analyses of randomised controlled trials (RCTs) that assessed the key questions listed above irrespective of language of publication. The key search terms were: very low birthweight or preterm AND (enteral feeding or enteral nutrition) AND (systematic review or meta-analysis). We checked the reference lists of the identified articles and published guidelines for systematic reviews not previously identified.

### Selection of reviews

Criteria for inclusion of reviews are shown in box 1. Reviews reporting interventions not currently widely available in sSA, such as use of donor breast milk,^13^ transpyloric feeding^14^ and specific nutritional supplements,^15–17^ were excluded. Two authors independently screened titles and abstracts to identify relevant reviews for full-text review. Disagreements were resolved by discussion involving a third author.

Box 1 **Criteria for inclusion of reviews**

**Participants**

- Neonates (babies aged <28 days)
- Birth weight less <1500 g and/or gestation <32 weeks

**Interventions**

- When to start enteral feeds:
  - Age <24 hours or ≥24 hours
- How to advance feeds:
  - Slow (15–20 mL/kg/day) versus fast (30–35 mL/kg/day)
- What to feed when mother’s milk is insufficient or not available:
  - Preterm formula versus standard infant formula
  - Unfortified versus fortified breastmilk
- How to feed:
  - Nasogastric versus orogastric tube versus cup and spoon feeding
  - Continuous versus bolus feeds
  - Gravity versus push feeds
- Interventions not currently widely available in sSA were excluded.

**Outcomes**

- Primary outcomes
  - Weight gain during hospital stay (g/kg/day or time to regain birth weight)
  - Time in days to achieve full enteral feeds (120 mL/kg/day)
- Secondary outcomes
  - Gain in length and head circumference during hospital stay
  - Frequency of necrotising enterocolitis, sepsis
  - Duration of hospital stay in days
  - Cognitive development
  - Mortality

**Reviews**

- Included: peer-reviewed systematic reviews of randomised clinical trials including cluster randomised or quasi-randomised trials
- Excluded: non-randomised designs

### Data extraction and management

One author extracted data from all selected reviews into a spreadsheet (Microsoft Excel) including number and settings of the included trials, total number and characteristics of participants, intervention(s) assessed, outcomes measured and major limitations. A second author crosschecked the extracted data for accuracy.

### Assessment of methodological quality of included reviews

Two authors independently assessed the quality of each review using the revised AMSTAR 2 (A Measurement Tool to Assess systematic Reviews) tool.^18^ Discrepancies were resolved by discussion and, if necessary, arbitration by a third author. The level of confidence in the findings of the reviews was assessed according to the number of critical and minor flaws in the methodology.^18^

### Outcomes

Primary outcomes were weight gain during hospital stay and time to achieve full enteral feeds (120 mL/kg/day). These were selected on the basis that they are recorded routinely in LMIC settings and are directly relevant to clinical practice. Secondary outcomes included growth, other important clinical outcomes such as sepsis, necrotising enterocolitis (NEC), death, duration of hospital stay and cognitive development (box 1).

### Data synthesis

We presented the findings for the primary and secondary outcomes for each intervention.^19^ We reported other outcome data in a narrative form. The relevance of the findings to feeding VLBW/very preterm infants in sSA was evaluated.

### Patient and public involvement

Patients and/or the public were not involved in the design, or conduct, or reporting, or dissemination plans of this research.

## Results

**Figure 1:**
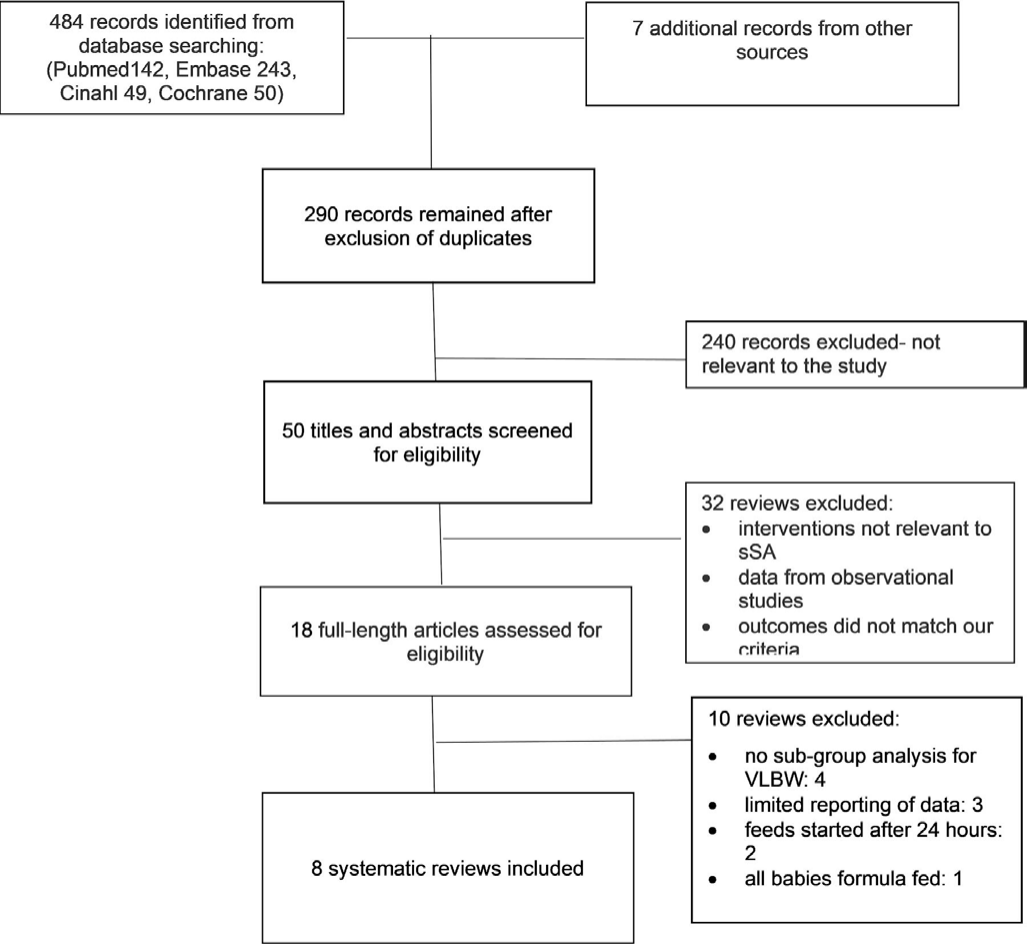
Flow diagram of selection of reviews. sSA, sub-Saharan Africa; VLBW, very low birth weight.

The search yielded 491 reviews (figure 1). After excluding duplicate and updated reviews, the titles and abstracts of 50 reviews were assessed for inclusion. The full texts of 18 reviews were screened of which 10 were excluded (see online supplementary file 1). Eight reviews met our inclusion criteria (table 1). The number of studies included in reviews ranged from 1 to 14 and the number of participants from 14 to 3753. Some of the findings from studies were pooled in a meta-analysis in four reviews.^20–23^ None of the reviews included studies from low-income countries including in sSA. Table 2 summarises the outcomes measured and estimates of size of effects of each intervention.

10.1136/bmjpo-2020-000724.supp1 Supplementary data

**Table 1:** Characteristics of included reviews

Review	Population	Intervention arm	Comparison arm	Outcomes reported
1. How to advance feeds				
Abiramalatha *et al* 24	Preterm (<37 weeks) or low birthweight (<2500 g) infants	High volume enteral feeds up to 300 mL/kg/day	Standard volume enteral feeds ≤200 mL/kg/day	Weight gain during hospital stay Feed intolerance Necrotising enterocolitis
Oddie *et al* 22	Enterally fed very preterm (<32 weeks) or VLBW infants	Faster advancement of enteral feeds	Advancement of enteral feeds no faster than 24 mL/kg (birth weight or current weight) per day	Feed intolerance Time to establish full enteral feeding Incidence of invasive infections Necrotising enterocolitis Duration of hospital stay Mortality
Alshaikh *et al* 23	VLBW (1000 to <1500 g) stable newborn infants	Enteral feeds started on day 1 at 80 mL/kg/day and increased by 20 mL/kg/day	Enteral feeds started on day 1 at ≤30 mL/kg/day and increased by 20 mL/kg/day	Time to regain birth weight Feed intolerance: time to establish full enteral feeds. Necrotising enterocolitis and sepsis Duration of hospital stay
2. What to feed				
Brown *et al* 20	Preterm (<37 weeks) and low birthweight (<2500 g) infants receiving breast milk in hospital	Fortification of breast milk with energy (carbohydrate or fat) and protein. Fortifiers could additionally include micronutrients and vitamins	Breast milk not fortified with energy or protein but can receive micronutrients and vitamins	Growth: weight, length, head circumference Length of hospital stay Feed intolerance Necrotising enterocolitis Bone mineralisation: serum alkaline phosphatase, bone mineral content Neurodevelopmental outcomes at 18 months: mental development index and psychomotor development index
Amissah *et al* 25	Preterm (<37 weeks) infants receiving enteral feeding of human milk in hospital	Human milk with additional fat supplementation	Human milk without additional fat supplementation	Growth: weight, length, head circumference
Amissah *et al* 26	Preterm (<37 weeks) infants receiving human milk in hospital	Human milk with additional carbohydrate supplementation	Human milk without additional carbohydrate supplementation	Weight at day 30 of age Duration of hospital stay Feed intolerance Necrotising enterocolitis
3. How to feed				
Watson *et al* 27	Preterm (<37 weeks) or low birthweight (<2500 g) infants receiving tube feeding	Nasal placement of feeding tubes	Oral placement of feeding tubes	Time to establish full tube feeds Time to regain birth weight Weight gain Time to independence from supplemental oxygen
Premji and Chessell^21^	VLBW infants with no history of feeding or feed intolerance and no congenital anomalies that might interfere with establishing enteral feeds	Continuous nasogastric feeding with human milk or infant formula	Intermittent bolus nasogastric feeding with human milk or infant formula	Time to establish full enteral feeds Time to establish full oral feeds Feed intolerance Days on TPN Time to regain birth weight Growth: weight, length, head circumference, triceps skinfold thickness Duration of hospital stay Days to discharge weight of 2040 g Days on mechanical ventilation Proven or probable necrotising enterocolitis Failure to complete protocol due to feed intolerance Apnoea

TPN, total parenteral nutrition; VLBW, very low birth weight (<1500 g).

**Table 2:** Findings from systematic reviews

Review/outcome	No of studies (no of babies)	Results MD or RR (95% CI)	Quality of evidence (GRADE assessment)
1. How to advance feeds			
*Abiramalatha et al* 24: *high volume versus standard volume feeds*			
Weight gain (g/kg/day)	1 (64)	MD 6.2 (2.71 to 9.69)	Low
Frequency of NEC	1 (64)	RR 1.03 (0.07 to 15.78)	Very low
Feed intolerance	1 (64)	RR 1.81 (0.89 to 3.67)	Low
Mortality	1 (64)	No death in any group	NR
*Oddie et al* 22: *slow versus faster rate or few advancement*			
Weight gain at discharge	1 (2804)	MD 0.0 (−0.08 to 0.08)	NR
Time to full feeds	8 (3551)	Longer time in slow advancement group. CI NR	NR
Gain in Head Circumference (HC) z score at discharge	1 (2804)	MD 0.0 (−0.13 to 0.13)	NR
Frequency of NEC	10 (3738)	RR 1.07 (CI 0.83 to 1.39)	Moderate
Frequency of sepsis	8 (3391)	RR 1.15 (1.00 to 1.32)	Low
Duration of hospital stay in days		2 trials reported longer duration in slow advancement group	NR
Mortality	9 (3553)	RR 1.15 (0.93 to 1.42)	Moderate
*Alshaikh et al* 23: *early total enteral feeds versus early partial enteral feeds*			
Time to regain birth weight	2 (149)	MD −1.15 days (−1.86 to −0.45)	Moderate (Jadad score)
Feed intolerance	3 (347)	RR 0.78 (0.38 to 1.59)	Moderate (Jadad score)
Time to full enteral feeds	2 (164)	MD −1.01 days (−1.36 to −0.66)	Moderate (Jadad score)
Frequency of NEC	4 (393)	RR 0.87 (0.19 to 3.98)	Moderate (Jadad score)
Incidence of late onset sepsis	3 (290)	RR 0.43 (0.30 to 0.61)	Moderate (Jadad score)
Duration of hospital stay	4 (393)	MD −1.35 days (−2.57 to −0.13)	Moderate (Jadad score)
2. What to feed			
*Brown et al* 20: *breast milk fortified with carbohydrate, fat and protein versus unfortified breast milk*			
Weight gain (g/kg/day)	5 (269)	MD 2.82 (1.83 to 3.80)	Low
Gain in length (cm/wk)	3 (189)	MD 0.21 (0.14 to 0.28)	Low
Gain in HC (cm/wk)	3 (189)	MD 0.11 (0.05 to 0.17)	Moderate
Frequency of NEC	7 (539)	RR 1.19 (0.49 to 2.88)	Low
Feed intolerance	5 (255)	RR 0.90 (0.54 to 1.49)	Low
Duration of hospital stay in weeks	2 (210)	MD 0.38 (−0.16 to 0.93)	Low
*Amissah et al* 25: *breast milk supplemented with fat versus unsupplemented breast milk*			
Weight gain (g/kg/day)	1 (14)	MD 0.60 (−2.4 to 3.6)	Very low
Length gain (cm/wk)	1 (14)	MD 0.10 (−0.08 to 0.3)	Very low
Head circumference gain (cm/wk)	1 (14)	MD 0.2 (−0.07 to 0.4)	Very low
Feed intolerance	1 (16)	RR 3.0 (0.1 to 64.3)	Very low
*Amissah et al* 26: *breast milk supplemented with prebiotic carbohydrate versus unsupplemented breast milk*			
Weight gain at age 30 days (g)	1 (75)	MD 160.4 (12.4 to 308.4)	Very low
Frequency of NEC	1 (75)	RR 0.2 (0.02 to 1.3)	Very low
Duration of hospital stay in days	1 (75)	Median (range) supplement versus control: 16 (9 to 45) versus 25 (11 to 80) p=0.004	Very low
3. How to feed			
*Watson and McGuire* 27: *nasal versus oral placement of feeding tube*			
Time to regain birth weight in days	1 (46)	MD 0.90 (−1.27 to 3.07)	NR ‘Study underpowered to exclude modest but plausible effect sizes’
Time to full feeds (days)	1 (46)	MD −2.7 (−11.9 to 6.5)	NR
*Premji and Chessell* 21: *continuous nasogastric milk versus intermittent bolus feeds*			
Weight gain (g/wk)	2 (106)	MD 6.27 (−1.28 to 13.81)	NR
Time to regain birth weight in days	3 (206)	MD −0.31 (−1.65 to 1.03)	NR
Time to full feeds in days	4 (229)	MD 1.82 (−0.44 to 4.08)	NR
Gain in length (cm/wk)	3 (159)	MD 0.07 (−0.04 to 0.18)	NR
Gain in HC (cm/wk)	2 (77)	MD −0.01 (−0.12 to 0.13)	NR
Frequency of proven NEC	4 (270)	RR 2.23 (0.58 to 8.57)	NR
Duration of hospital stay in days	1 (82)	MD −1.00 (−8.62 to 6.62)	NR

GRADE, Grading of Recommendation Assessment Development and Evaluation; MD, mean difference; NEC, necrotising enterocolitis; NR, not reported.

### When to start feeds

We did not find any reviews comparing the effects of starting feeds before or after 24 hours of life.

### How to advance enteral feeds

One review that assessed increasing feeds up to 300 mL/kg/day versus 200 mL/kg/day included 1 study which recruited 64 babies. Weight gain was greater in the high-volume group with no increase in feeding intolerance, NEC or mortality. The authors assessed the quality of evidence as low to very low because of heterogeneity and risk of bias due to lack of blinding.^24^

One review assessed daily increments of less than 24 mL/kg compared with 30–40 mL/kg. There were 10 studies with a total of 3753 participants. Rate of feed advancement was not associated with weight gain, frequency of NEC or mortality. Slow advancement of feeds may have resulted in a longer time to achieve full feeds, longer hospital stay and a higher risk of sepsis. The authors downgraded the quality of evidence to moderate because there was a lack of blinding in most studies.^22^

One review that assessed advancing feeds to 80 mL/kg by the end of the first day compared with 20–30 mL/kg included 4 randomised trials and a total of 393 babies.^23^ In clinically stable babies, those who had higher volume feeds had lower rates of late-onset sepsis and shorter time to full enteral feeds; these effects were statistically significant in meta-analysis and there was no significant heterogeneity. Higher volume feeds also reduced time to regain birth weight but with significant heterogeneity between the trials. Only two trials reported duration of hospital stay and these trials had significant heterogeneity. The quality of the evidence from the trials was rated as 2 to 3 (low to adequate) on the Jadad scale.

### What to feed when mother’s milk is insufficient or not available

There were no reviews comparing the effect of preterm versus standard term formula.

One review that assessed the effect of fortification of human milk with both energy and protein included 14 trials (13 RCTs and one quasi-RCT) and a total of 1071 participants.^20^ Meta-analysis showed a higher weight, length and head circumference at discharge, 12 and 18 months in the fortified group. There was no significant effect on neurodevelopment, NEC, feed intolerance or length of hospital stay. The review authors downgraded the quality of evidence to low as there was significant heterogeneity possibly due to the use of different feeds: some studies used mother’s milk only, some mother’s milk and donor milk (when mother’s milk was insufficient) and others mother’s milk and formula.

A systematic review assessed the effect of addition of fat to human milk feeds. The review included 1 study with only 14 babies; there was no evidence that the addition of fat improved in-hospital growth or affected tolerance of feeds.^25^ The quality of evidence was very low due to incomplete description of the methodology and small sample size.

A review that assessed the effect of supplementation of human milk with short‐chain galacto‐oligosaccharides/long‐chain fructo‐oligosaccharides included 1 study with 75 participants. There was a significant increase in weight in the supplemented group and decrease in the length of hospital stay but no effect on the incidence of NEC or sepsis.^26^ The review authors downgraded the quality of evidence to very low because of a significant risk of bias and the uncertainty resulting from the small sample size.

### How to feed

#### Nasogastric versus orogastric tube versus cup and spoon feeding

A review that assessed nasal versus oral placement of enteral feeding tubes and included 1 RCT with 46 infants found insufficient evidence that the placement of enteral feeding tubes affected time to full enteral feeds, growth and feed intolerance. The review authors assessed the quality of evidence as low due to lack of blinding and failure to report all important outcomes.^27^ There was no review assessing cup and spoon feeding in VLBW babies.

#### Continuous versus bolus feeds

One review that assessed continuous nasogastric versus intermittent bolus feeds included 7 trials and 511 babies. There were no significant differences in growth, length of hospital stay or incidence of NEC. The review authors concluded that the benefits and risks of continuous and intermittent feeding could not be assessed from current RCTs and called for future studies to be conducted with consistent feeding protocols and definitions of feed intolerance.^21^

#### Gravity versus push feeds

We found no reviews assessing gravity versus push feeds.

### Methodological quality of the included reviews

We have reported the AMSTAR2 assessments in table 3.

**Table 3:** AMSTAR2 ratings of included systematic reviews

Review	AMSTAR2 rating																Confidence in findings of review
	Q1	Q2	Q3	Q4	Q5	Q6	Q7	Q8	Q9	Q10	Q11	Q12	Q13	Q14	Q15	Q16	
Abiramalatha *et al* 24	Y	N	N	Y	Y	Y	Y	Y	Y	N	NA	NA	Y	Y	NA	Y	Moderate
Alshaikh *et al* 23	Y	N	N	Y	Y	Y	N	Y	Y	N	Y	N	N	N	Y	Y	Critically low
Amissah *et al* 25	Y	Y	N	Y	Y	Y	Y	Y	Y	N	NA	NA	Y	Y	NA	N	Moderate
Amissah *et al* 26	Y	Y	N	Y	Y	Y	Y	Y	Y	Y	NA	NA	Y	Y	NA	Y	High
Brown *et al* 20	Y	N	N	Y	Y	Y	Y	Y	Y	N	Y	Y	Y	Y	N	Y	Moderate
Oddie *et al* 22	Y	N	N	Y	Y	Y	Y	Y	Y	N	Y	Y	Y	Y	Y	Y	Moderate
Premji and Chessell^21^	Y	PY	N	Y	Y	Y	Y	PY	Y	N	Y	Y	Y	Y	N	Y	Moderate
Watson and McGuire^27^	Y	N	N	Y	Y	Y	Y	Y	Y	N	NA	NA	Y	Y	NA	Y	Moderate

AMSTAR2 ratings are as follows.

1. Did the research questions and inclusion criteria for the review include the components of PICO?

2. Did the report of the review contain an explicit statement that the review methods were established prior to the conduct of the review and did the report justify any significant deviations from the protocol?

3. Did the review authors explain their selection of the study designs for inclusion in the review?

4. Did the review authors use a comprehensive literature search strategy?

5. Did the review authors perform study selection in duplicate?

6. Did the review authors perform data extraction in duplicate?

7. Did the review authors provide a list of excluded studies and justify the exclusions?

8. Did the review authors describe the included studies in adequate detail?

9. Did the review authors use a satisfactory technique for assessing the risk of bias (RoB) in individual studies that were included in the review?

10. Did the review authors report on the sources of funding for the studies included in the review?

11. If meta-analysis was performed, did the review authors use appropriate methods for statistical combination of results?

12. If meta-analysis was performed, did the review authors assess the potential impact of RoB in individual studies on the results of the meta-analysis or other evidence synthesis?

13. Did the review authors account for RoB in primary studies when interpreting/discussing the results of the review?

14. Did the review authors provide a satisfactory explanation for, and discussion of, any heterogeneity observed in the results of the review?

15. If they performed quantitative synthesis did the review authors carry out an adequate investigation of publication bias (small study bias) and discuss its likely impact on the results of the review?

16. Did the review authors report any potential sources of conflict of interest, including any funding they received for conducting the review?

n, no; NA, not applicable; PICO, Population Intervention Comparison Outcome; PY, partial yes; Y, yes.

PICO components regarding the research questions and inclusion criteria (Q1) were stated in all reviews. Only two reviews stated clearly that the review methods had been established prior to the conduct of the review and justified any significant deviations in methodology where appropriate.^25 26^ Although standard Cochrane methodology includes publication of a protocol (Q2) and assessment of the likelihood and impact of publication bias (Q15), we downgraded our quality assessment for Cochrane reviews if these elements were not reported. Methods for searching the literature (Q4), selecting studies for inclusion (Q5), extracting data (Q6) and assessing risk of bias (Q9) were done well in all studies. Only one study reported on the sources of funding for the studies (Q10).^26^ All reviews except one^25^ reported potential sources of conlfict of interest. The only review that was not a Cochrane review had several methodological flaws which led to a critically low level of confidence in its findings.^23^ Overall, our confidence in the findings of the reviews was critically low in 1, moderate in 6 and high in only 1.

## Discussion

This overview demonstrates that there is a severe lack of evidence to inform basic feeding practices in this highly vulnerable group of infants in low-resource settings in sSA. We found evidence supporting starting feeds at a higher volume, faster advancement of feeds, fortification of breast milk with energy and protein or prebiotics. However, we found only eight systematic reviews which included small numbers of trials, some of which had few participants. Quality of evidence as assessed by the review authors ranged from very low to moderate. An inherent difficulty in evaluating some feeding approaches is blinding of trial arms. Furthermore, our confidence in the findings was high for only one review. No reviews of RCTs in VLBW babies assessed important issues such as the use of preterm formula, cup and spoon feeding and gravity versus push feeds. Finally, none of the reviews included trials done in the low-income countries of sSA.

The strengths of this review are that it collates the evidence of RCTs of feeding interventions for highly vulnerable newborns that are feasible in low-resource settings. It appraises the quality of reviews using a validated measure (AMSTAR2) and updates and expands on the findings of the evidence review commissioned by the WHO published in 2011.^9^ A weakness in our review of the evidence that informs feeding practices is that we limited our search to systematic reviews and have not considered other forms of evidence such as RCTs not included in reviews.

Human donor milk has been recommended by the WHO as the best alternative when mother’s own milk is not available but there are few human milk banks operating in sSA.^9^ The practice of wet nursing and informal milk sharing has been carried out in many societies in Africa but we did not find any systematic reviews in hospital settings. In recent years, these practices have been discouraged due to fears of HIV transmission but qualitative studies in sSA have explored parents’ and healthcare workers’ attitudes to using screened and pasteurised donor human milk through facility-based human milk banks.^28 29^

Low-income countries lack resources to provide parenteral nutrition to VLBW/very preterm babies, particularly in the public health facilities. Hence, the focus is on achieving full enteral feeds as soon as possible, with the potential benefits of reducing length of hospital stay and exposure to hospital-acquired infection, as well as enhancing brain growth. Initiation of enteral feeding was previously delayed through fears of adverse effects on the immature gut, and we did not find any systematic reviews comparing initiation in the first 24 hours with later introduction. A systematic review, which was excluded because the cut-off for early versus delayed initiation was 4 days, found no evidence of decreased risk of NEC if feeds were delayed, but did find a delay in achieving full enteral feeds.^30^ The definition of full enteral feeds was not consistent within and between reviews with some studies reporting milk volumes and others kilocalories consumed. Some studies specified for how long feeds were tolerated before meeting the definition. This makes direct comparisons of this outcome measure impossible.

The fetus swallows up to 250 mL/kg/day of amniotic fluid that contains trophic factors and provides 10% of daily nutrient requirements suggesting that the newborn gut may tolerate higher fluid volumes than have been previously given.^31^ Faster rates of feed advancement (30–40 mL/kg/day),^22^ and higher volumes of feeds (up to 300 mL/kg/day)^24^ had some evidence of benefit in terms of higher weight gain, shorter length of hospital stay,^22^ shorter time to full enteral feeds^22^ and reduced incidence of late-onset sepsis^22^ without increasing the risk of NEC. Further trials of these approaches in low-income settings are warranted.

Multinutrient and prebiotic fortification of breast milk shows significant short-term effects but, apart from limited evidence of a lack of effect on psychomotor development at 18 months, evidence on longer term effect is lacking. Fortifying breast milk with fat alone was not shown to confer any benefit but these results were from a small study and, therefore, the evidence was limited. Fortifying feeds with fat may improve longer term outcomes, given the theoretical benefit of fats on cognition and neurodevelopment.^32^ Larger trials in LMIC settings, designed and powered to measure the long-term effects of breast milk fortifiers on growth and development, the incidence of adverse events as well as cost effectiveness are desirable. However, research results may not be applicable until multinutrient fortifiers become readily available and affordable in LMICs. A pragmatic alternative would be to test the effects of term formula milk powder for supplementation of calories and macronutrients with due attention to possible adverse effects.

## Conclusion

Evidence that informs basic feeding practices of VLBW/very preterm infants in low-resource settings in sSA is poor. There is an urgent need for trials that evaluate pragmatic, inexpensive, low technology and sustainable feeding interventions. Follow-up is needed to include longer term growth and neurodevelopmental outcomes.

## Supplementary Material

Author's manuscript
