# Supplementary material for: Evidence that informs feeding practices in very low birthweight and very preterm infants in sub-Saharan Africa: an overview of systematic reviews
Source: BMJ Paediatr Open. 2020 Aug 11;4(1):e000724. doi: 10.1136/bmjpo-2020-000724 (PMC7422638; doi:10.1136/bmjpo-2020-000724)
Supplement: Supplementary data [file bmjpo-2020-000724supp001.pdf]

Characteristics of Excluded Reviews

Review	Reason for exclusion
Flint 2016[1]	Included term babies. No sub-group analysis for VLBW infants
Morgan 2013[2]	Trophic feeds did not start before 24 hours
Morgan 2014[3]	Enteral feeds did not start before 24 hours
Richards 2014[4]	No RCTs on VLBW infants
Dawson 2012[5]	No sub-group analysis for VLBW infants
Moon 2016[6]	All babies formula fed
Kwok 2019[7]	Limited reporting of growth data
Civardi 2015[8]	Meeting abstract- limited reporting of data
Amissah 2018[9]	No sub-group analysis for VLBW infants
De Pipaon 2019[10]	Meeting abstract – limited reporting of data

References

1. Flint A, New K, Davies MW. Cup feeding versus other forms of supplemental enteral feeding for newborn infants unable to fully breastfeed. *Cochrane Database Syst Rev* 2016(8):CD005092. doi: 10.1002/14651858.CD005092.pub3 [published Online First: 2016/09/01]

2. Morgan J, Bombell S, McGuire W. Early trophic feeding versus enteral fasting for very preterm or very low birth weight infants. *Cochrane Database Syst Rev* 2013(3):CD000504. doi: 10.1002/14651858.CD000504.pub4 [published Online First: 2013/04/02]

3. Morgan J, Young L, McGuire W. Delayed introduction of progressive enteral feeds to prevent necrotising enterocolitis in very low birth weight infants. *Cochrane Database Syst Rev* 2014(12):CD001970. doi: 10.1002/14651858.CD001970.pub5 [published Online First: 2014/12/02]

4. Richards R, Foster JP, Psaila K. Continuous versus bolus intragastric tube feeding for preterm and low birth weight infants with gastro-oesophageal reflux disease. *Cochrane Database Syst Rev* 2014(7):CD009719. doi: 10.1002/14651858.CD009719.pub2 [published Online First: 2014/07/18]

5. Dawson JA, Summan R, Badawi N, et al. Push versus gravity for intermittent bolus gavage tube feeding of premature and low birth weight infants. *Cochrane Database Syst Rev* 2012;11:CD005249. doi: 10.1002/14651858.CD005249.pub2 [published Online First: 2012/11/16]

6. Moon K, Rao SC, Schulzke SM, et al. Longchain polyunsaturated fatty acid supplementation in preterm infants. *Cochrane Database Syst Rev* 2016;12:CD000375. doi: 10.1002/14651858.CD000375.pub5 [published Online First: 2016/12/21]

7. Kwok TC, Dorling J, Gale C. Early enteral feeding in preterm infants. *Semin Perinatol* 2019;43(7):151159. doi: 10.1053/j.semperi.2019.06.007 [published Online First: 2019/08/25]

8. Civardi E. Trophic feeding for very preterm or very low birth weight infants. *Italian Journal of Pediatrics* 2015;41(Suppl 1):A3.

9. Amissah EA, Brown J, Harding JE. Protein supplementation of human milk for promoting growth in preterm infants. *Cochrane Database Syst Rev* 2018;6:CD000433. doi: 10.1002/14651858.CD000433.pub2 [published Online First: 2018/06/23]

10. De Pipaon M.S. LMR, Couce M.L., Sanz-Valero J. Effect of long-chain polyunsaturated fatty acid supplementation of preterm infants on infection. A systematic review and meta-analysis. *J Pediatr Gastroenterol Nutr* 2019;68(Supplement 1):1005-06.
