# Supplementary material for: Evidence that informs feeding practices in very low birthweight and very preterm infants in sub-Saharan Africa: an overview of systematic reviews
Source: BMJ Paediatr Open. 2020 Aug 11;4(1):e000724. doi: 10.1136/bmjpo-2020-000724 (PMC7422638; doi:10.1136/bmjpo-2020-000724)
Supplement: Author's manuscript [file bmjpo-2020-000724.draft_revisions.pdf]

BMJ Paediatrics Open

BMJ Paediatrics Open is committed to open peer review. As part of this commitment we make the peer review history of every article we publish publicly available.

When an article is published we post the peer reviewers' comments and the authors' responses online. We also post the versions of the paper that were used during peer review. These are the versions that the peer review comments apply to.

The versions of the paper that follow are the versions that were submitted during the peer review process. They are not the versions of record or the final published versions. They should not be cited or distributed as the published version of this manuscript.

BMJ Paediatrics Open is an open access journal and the full, final, typeset and author-corrected version of record of the manuscript is available on our site with no access controls, subscription charges or pay-per-view fees (http://bmjpaedsopen.bmj.com).

If you have any questions on BMJ Paediatrics Open's open peer review process please email info.bmjpo@bmj.com

BMJ Paediatrics Open

Evidence that informs feeding practices in very low birth weight and very preterm infants in sub-Saharan Africa: An overview of systematic reviews

Journal:	*BMJ Paediatrics Open*
Manuscript ID	bmjpo-2020-000724
Article Type:	Original research
Date Submitted by the Author:	04-May-2020

Complete List of Authors:	Akindolire, Abimbole; University of Ibadan College of Medicine<br>Talbert, Alison; Centre for Geographic Medicine Research Coast<br>Sinha, Ian; Alder Hey Children's NHS Foundation Trust, Respiratory Medicine<br>Embleton, Nick; Newcastle University<br>Allen, Stephen; Liverpool School of Tropical Medicine

Keywords:	Neonatology

SCHOLARONE™
Manuscripts

I, the Submitting Author has the right to grant and does grant on behalf of all authors of the Work (as defined in the below author licence), an exclusive licence and/or a non-exclusive licence for contributions from authors who are: i) UK Crown employees; ii) where BMJ has agreed a CC-BY licence shall apply, and/or iii) in accordance with the terms applicable for US Federal Government officers or employees acting as part of their official duties; on a worldwide, perpetual, irrevocable, royalty-free basis to BMJ Publishing Group Ltd ("BMJ") its licensees and where the relevant Journal is co-owned by BMJ to the co-owners of the Journal, to publish the Work in this journal and any other BMJ products and to exploit all rights, as set out in our licence.

The Submitting Author accepts and understands that any supply made under these terms is made by BMJ to the Submitting Author unless you are acting as an employee on behalf of your employer or a postgraduate student of an affiliated institution which is paying any applicable article publishing charge ("APC") for Open Access articles. Where the Submitting Author wishes to make the Work available on an Open Access basis (and intends to pay the relevant APC), the terms of reuse of such Open Access shall be governed by a Creative Commons licence – details of these licences and which Creative Commons licence will apply to this Work are set out in our licence referred to above.

Other than as permitted in any relevant BMJ Author's Self Archiving Policies, I confirm this Work has not been accepted for publication elsewhere, is not being considered for publication elsewhere and does not duplicate material already published. I confirm all authors consent to publication of this Work and authorise the granting of this licence.

Confidential: For Review Only

Review title

Evidence that informs feeding practices in very low birthweight and very preterm infants in sub-Saharan Africa: An overview of systematic reviews

Abimbola E Akindolire MB BS,[1*] Alison W Talbert MRCP,[2*] Ian Sinha PhD,[3] Nicholas D Embleton MD,[4] Stephen J Allen MD [3,5] on behalf of the Neonatal Nutrition Network (NeoNuNet)

*Joint first authors

Corresponding author:

Dr. Alison Talbert

KEMRI-Wellcome Trust Research Programme

P.O. Box 230

Kilifi 80108

Kenya

Phone: +254 729 218993

Email: atalbert@kemri-wellcome.org

Affiliations

1. University College Hospital, Ibadan, Nigeria
2. KEMRI-Wellcome Trust Research Programme, Kilifi, Kenya
3. Alder Hey Children's Hospital NHS Trust, Liverpool, UK
4. Newcastle University, Newcastle, UK
5. Liverpool School of Tropical Medicine, Liverpool, UK

Confidential: For Review Only

Neonatal Nutrition Network members: Olusegun Akinyinka (College of Medicine, University of Ibadan, Nigeria); Dominic D Omuru (Maitama District Hospital, Abuja, Nigeria); Chinyere Ezeaka (Lagos University Teaching Hospital, Nigeria); Beatrice N Ezenwa (Lagos University Teaching Hospital, Nigeria); Iretiola B Fajolu (Lagos University Teaching Hospital, Nigeria); Zainab O Imam (Massey St. Children's Hospital, Lagos, Nigeria); Martha K Mwangome (KEMRI Wellcome Trust Research Programme, Kilifi, Kenya); Alison W Talbert (KEMRI Wellcome Trust Research Programme, Kilifi, Kenya); Pauline EA Andang'o (Maseno University, Kenya); Walter Otieno (Jaramogi Oginga Odinga Teaching and Referral Hospital, Kisumu, Kenya & Maseno University, Kenya); Grace M Nalwa (Jaramogi Oginga Odinga Teaching and Referral Hospital, Kisumu, Kenya & Maseno University, Kenya); Ian Sinha (Alder Hey Children's Hospital, Liverpool, UK); Janneke van de Wijgert (University of Liverpool, Liverpool, UK); Melissa Gladstone (University of Liverpool, Liverpool, UK); Kevin Mortimer (Liverpool School of Tropical Medicine, Liverpool, UK); Graham Devereux (Liverpool School of Tropical Medicine, Liverpool, UK); Ismaela Abubakar (Liverpool School of Tropical Medicine, Liverpool, UK); Stephen Turner (University of Aberdeen, Aberdeen, UK); Nicholas Embleton (Newcastle University, Newcastle, UK); Helen M Nabwera (Liverpool School of Tropical Medicine, Liverpool, UK); Stephen J Allen (Liverpool School of Tropical Medicine, Liverpool, UK); Isa Abdulkadir (Ahmadu Bello University, Zaria, Nigeria); Olukemi O Tongo (College of Medicine, University of Ibadan, Nigeria); Abimbola E Akindolire (College of Medicine, University of Ibadan, Nigeria).

Manuscript word count 3084

Confidential: For Review Only

Abstract

Background Optimal feeding of very low birthweight (VLBW <1500g)/very preterm (gestation <32 weeks) infants in resource-limited settings in sub-Saharan Africa (sSA) is critical to reducing high mortality and poor outcomes.

Objective To review evidence on feeding of VLBW/very preterm infants relevant to sSA.

Methods We searched the Cochrane Database of Systematic Reviews, EMBASE, PUBMED and CINAHL from inception to July 2019 to identify reviews of randomised and quasi-randomised controlled trials of feeding VLBW/very preterm infants. We focused on interventions that are readily available in sSA. Primary outcomes were weight gain during hospital stay and time to achieve full enteral feeds (120 mls/kg/day). Secondary outcomes were growth, common morbidities, mortality, duration of hospital stay and cognitive development. Quality of evidence (QOE) was assessed using the Measurement Tool to Assess Systematic Reviews (AMSTAR2).

Results Eight systematic reviews were included. Higher feed volume of day 1 (80 ml/kg) reduced late-onset sepsis and time to full enteral feeds, and higher feed volume (up to 300 mls/kg/day) improved weight gain without adverse events (QOE: low-moderate). Rapid advancement of feeds (30-40 ml/kg/day) was not associated with harm. Breast milk fortification with energy and protein increased growth and with prebiotics increased growth and reduced duration of admission (QOE: low -very low) and did not result in harm. Evidence regarding feeding tube placement and continuous versus bolus feeds was insufficient to draw conclusions. We found no reviews meeting our selection criteria regarding when to start feeds, use of preterm

Confidential: For Review Only

formula, cup-and-spoon feeding or gravity versus push feeds and none of the reviews included trials from low-income countries of sSA.

Conclusions The evidence base informing feeding of VLBW/very preterm babies in resource-limited settings in sSA is extremely limited. Pragmatic studies are needed to generate evidence to guide management and improve outcomes for these highly vulnerable infants.

Protocol registration

http://www.crd.york.ac.uk/PROSPERO/display_record.php?ID=CRD42019140204

Abstract 300 words

For Review Only

What is known already on this topic?

- Although survival is improving, mortality and outcomes for VLBW/very preterm infants in resource-limited settings in sSA remain poor.
- Optimising feeding is critical to improving survival, healthy growth and development and reducing morbidity.
- International and national protocols inform the feeding of VLBW/very preterm infants but evidence relevant to resource-limited settings is limited.

What this study adds

- No trials of pragmatic feeding interventions have been conducted in resource-limited settings in sSA.
- Higher feed volumes, rapid advancement of feeds and fortification of breast milk may be beneficial in achieving full feeds, improving growth and reducing morbidity without adverse effects.
- Trials of pragmatic feeding protocols in resource-limited settings are needed urgently to better inform clinical practice.

Background

Worldwide about 20 million babies are born low birth weight (birth weight (BW) <2500g) annually and 60% of these births occur in sub-Saharan Africa (sSA) and South Asia.[1] An increasing number of very low birth weight (VLBW; BW<1500g) babies are surviving till discharge in low and middle income countries (LMICs).[2] Optimal nutrition is key for survival, prevention of adverse events such as sepsis and reducing the length of the hospital stay.[3] In addition, there is strong evidence that early nutrition impacts on immune and respiratory function and longer term cognitive outcome as well as a range of chronic conditions such as diabetes and cardiovascular disease in adulthood which place a substantial burden on healthcare systems.[3-5]

Most very preterm infants (gestation <32 weeks) have yet to develop coordinated oromotor skills of sucking, swallowing and breathing that allow safe breastfeeding and, therefore, other methods of feeding are necessary. Uncertainty about gestational age due to a lack of reliable first trimester ultrasound scan is common in LMICs and birthweight is often used to identify infants who are preterm, growth-retarded or both.[6] In most neonatal units (NNUs) in LMICs, parenteral nutrition is not available.[7] Trials on enteral feeding interventions have been done mainly in high and middle-income countries. The generalisability of their findings to feeding VLBW infants in most of sSA is difficult to assess due to multiple differences in maternal, pregnancy, delivery, neonatal and environmental factors and also the levels of care and clinical monitoring provided in high versus low-middle income countries.[8-10]

Although internationally agreed guidelines on optimal feeding of low birthweight infants in LMICs, including some recommendations for VLBW infants, were published by the World Health Organization in 2011, these lacked a strong evidence-base especially

Confidential: For Review Only

for LMIC settings.[7] The aim of our study was to review the evidence for pragmatic, low cost interventions to optimise the feeding of VLBW/very preterm infants in low-resource settings in sSA. We systematically searched for evidence on when to start enteral feeds; how to advance feeds; what to feed when mother's own milk is insufficient or not available and how to feed.

Methods and analysis

Protocol registration

We registered the overview protocol prospectively on the PROSPERO International Prospective Register of Systematic Reviews (http://www.crd.york.ac.uk/PROSPERO/display_record.php?ID=CRD42019140204)

Patient and public involvement

There was no patient or public involvement in the planning or execution of this overview.

Search strategy

We searched the Cochrane Database of Systematic reviews, EMBASE, PUBMED and CINAHL from inception to July 2019 for systematic reviews and meta-analyses of randomised controlled trials (RCTs) that assessed the key questions listed above irrespective of language of publication. The key search terms were: very low birthweight or preterm AND (enteral feeding or enteral nutrition) AND (systematic review or meta-analysis). We checked the reference lists of the identified articles and published guidelines for systematic reviews not previously identified.

Selection of reviews

Confidential: For Review Only

Table 1: Criteria for inclusion of reviews

Participants<br>• Neonates (babies aged < 28 days)<br>• Birth weight less <1500g and/or gestation <32 weeks
Interventions<br>• When to start enteral feeds:<br>○ Age <24 hours or ≥24 hours<br>• How to advance feeds:<br>○ Slow (15-20 mL/kg/day) vs. fast (30-35 mL/kg/day)<br>• What to feed when mother's milk is insufficient or not available:<br>○ Preterm formula vs. standard infant formula<br>○ Unfortified vs. fortified breastmilk<br>• How to feed:<br>○ Nasogastric vs. orogastric tube vs. cup and spoon feeding<br>○ Continuous vs. bolus feeds<br>○ Gravity vs. push feeds<br>Interventions not currently widely available in sSA were excluded
Outcomes<br>• Primary outcomes<br>○ Weight gain during hospital stay (g/kg/day or time to regain birthweight)<br>○ Time in days to achieve full enteral feeds (120 mL/kg/day)<br>• Secondary outcomes<br>○ Gain in length and head circumference during hospital stay<br>○ Frequency of necrotising enterocolitis, sepsis<br>○ Duration of hospital stay in days<br>○ Cognitive development<br>○ Mortality
Reviews<br>• Included: peer reviewed systematic reviews of randomised clinical trials (RCTs) including cluster randomised or quasi-randomised trials<br>• Excluded: non-randomised designs

Criteria for inclusion of reviews are shown in Table 1. Reviews reporting interventions not currently widely available in sSA, such as use of donor breast milk,[11] transpyloric feeding,[12] and specific nutritional supplements[13-15] were excluded. Two authors independently screened titles and abstracts to identify relevant reviews for full-text review. Disagreements were resolved by discussion involving a third author.

Data extraction and management

Confidential: For Review Only

One author extracted data from all selected reviews into a spreadsheet (Microsoft Excel) including number and settings of the included trials, total number and characteristics of participants, intervention(s) assessed, outcomes measured and major limitations. A second author crosschecked the extracted data for accuracy.

Assessment of methodological quality of included reviews

Two authors independently assessed the quality of each review using the revised AMSTAR 2 (A Measurement Tool to Assess systematic Reviews) tool.[16] Discrepancies were resolved by discussion and, if necessary, arbitration by a third author. The level of confidence in the findings of the reviews was assessed according to the number of critical and minor flaws in the methodology.[16]

Outcomes

Primary outcomes were weight gain during hospital stay and time to achieve full enteral feeds (120 mls/kg/day). These were selected on the basis that they are recorded routinely in LMIC settings and are directly relevant to clinical practice. Secondary outcomes included growth, other important clinical outcomes such as sepsis, necrotising enterocolitis (NEC), death, duration of hospital stay and cognitive development (Table 1).

Data synthesis

We presented the findings for the primary and secondary outcomes for each intervention.[17] We reported other outcome data in a narrative form. The relevance of the findings to feeding VLBW/very preterm infants in sSA was evaluated.

Results

The search yielded 491 reviews (Figure 1). After excluding duplicate and updated reviews, the titles and abstracts of 50 reviews were assessed for inclusion. The full texts of 18 reviews were screened of which 10 were excluded (see Supplementary File 1). Eight reviews met our inclusion criteria (Table 2). The number of studies included in reviews ranged from 1 to 14 and the number of participants from 14 to 3753. Some of the findings from studies were pooled in a meta-analysis in 4 reviews.[18-21] None of the reviews included studies from low income countries including in sSA. Table 3 summarizes the outcomes measured and estimates of size of effects of each intervention.

Confidential: For Review Only

Table 2: Characteristics of included reviews

Review	Population	Intervention arm	Comparison arm	Outcomes reported
**1. How to advance feeds**				
Abiramalatha 2017	Preterm (<37 weeks) or low birth weight (<2500g) infants	High volume enteral feeds up to 300mL/kg/day	Standard volume enteral feeds ≤200mL/kg/day	• Weight gain during hospital stay<br>• Feed intolerance<br>• Necrotizing enterocolitis
Oddie 2017	Enterally fed very preterm (<32 weeks) or VLBW infants	Faster advancement of enteral feeds	Advancement of enteral feeds no faster than 24mL/kg (birth weight or current weight) per day	• Feed intolerance<br>• Time to establish full enteral feeding<br>• Incidence of invasive infections<br>• Necrotizing enterocolitis<br>• Duration of hospital stay<br>• Mortality
Alshaikh 2019	VLBW (1000-<1500g) stable newborn infants	Enteral feeds started on day 1 at 80mL/kg/day and increased by 20mL/kg/day	Enteral feeds started on day 1 at ≤30mL/kg/day and increased by 20mL/kg/day	• Time to regain birth weight<br>• Feed intolerance: time to establish full enteral feeds.<br>• Necrotizing enterocolitis and sepsis<br>• Duration of hospital stay
**2. What to feed**				
Brown 2016	Preterm (<37 weeks) and low birth weight (<2500g) infants receiving breast milk in hospital	Fortification of breast milk with energy (carbohydrate or fat) and protein. Fortifiers could additionally include micronutrients and vitamins	Breast milk not fortified with energy or protein but can receive micronutrients and vitamins	• Growth: weight, length, head circumference<br>• Length of hospital stay<br>• Feed intolerance<br>• Necrotizing enterocolitis<br>• Bone mineralization: serum alkaline phosphatase, bone mineral content

Confidential: For Review Only

				• Neurodevelopmental outcomes at 18 months: mental development index and psychomotor development index
Amissah 2018a	Preterm (<37 weeks) infants receiving enteral feeding of human milk in hospital	Human milk with additional fat supplementation	Human milk without additional fat supplementation	• Growth: weight, length, head circumference
Amissah 2018b	Preterm (<37 weeks) infants receiving human milk in hospital	Human milk with additional carbohydrate supplementation	Human milk without additional carbohydrate supplementation	• Weight at day 30 of age<br>• Duration of hospital stay<br>• Feed intolerance<br>• Necrotizing enterocolitis
**3. How to feed**				
Watson 2013	Preterm (<37 weeks) or low birth weight (<2500g) infants receiving tube feeding	Nasal placement of feeding tubes	Oral placement of feeding tubes	• Time to establish full tube feeds<br>• Time to regain birth weight<br>• Weight gain<br>• Time to independence from supplemental oxygen
Premji 2011	VLBW infants with no prior history of feeding or feed intolerance and no congenital anomalies that might interfere with establishing enteral feeds	Continuous nasogastric feeding with human milk or infant formula	Intermittent bolus nasogastric feeding with human milk or infant formula	• Time to establish full enteral feeds<br>• Time to establish full oral feeds<br>• Feed intolerance<br>• Days on TPN<br>• Time to regain birth weight<br>• Growth: weight, length, head circumference, triceps skinfold thickness<br>• Duration of hospital stay<br>• Days to discharge weight of 2040g<br>• Days on mechanical ventilation<br>• Proven or probable necrotizing enterocolitis

Confidential: For Review Only

				• Failure to complete protocol due to feed intolerance<br>• Apnoea

Notes: VLBW = very low birthweight (<1500g); TPN = total parenteral nutrition

Confidential: For Review Only

Table 3: Findings from systematic reviews

**Review/Outcome**	**No. of studies (no. of babies)**	**Results** MD or RR (95% CI)	**Quality of evidence** (GRADE assessment)
**1. How to advance feeds**			
Abiramalatha 2017: High volume vs standard volume feeds			
Weight gain (g/kg/day)	1 (64)	MD 6.2 (2.71 to 9.69)	Low
Frequency of NEC	1 (64)	RR 1.03 (0.07 to 15.78)	Very low
Feed intolerance	1 (64)	RR 1.81 (0.89 to 3.67)	Low
Mortality	1 (64)	No death in any group	NR
Oddie 2017: Slow vs faster rate or few advancement			
Weight gain at discharge	1 (2804)	MD 0.0 (-0.08 to 0.08)	NR
Time to full feeds	8 (3551)	Longer time in slow advancement group. CI NR	NR
Gain in HC z score at discharge	1 (2804)	MD 0.0 (-0.13 to 0.13)	NR
Frequency of NEC	10 (3738)	RR 1.07 (CI 0.83 to 1.39)	Moderate
Frequency of sepsis	8 (3391)	RR 1.15 (1.00 to 1.32)	Low
Duration of hospital stay in days		2 trials reported longer duration in slow advancement group	NR
Mortality	9(3553)	RR 1.15 (0.93 to 1.42)	Moderate
Alshaikh 2019: Early total enteral feeds vs early partial enteral feeds			
Time to regain birth weight	2 (149)	MD -1.15 days (-1.86 to -0.45)	Moderate (Jadad score)
Feed intolerance	3 (347)	RR 0.78 (0.38 to 1.59)	Moderate (Jadad score)
Time to full enteral feeds	2 (164)	MD -1.01 days (-1.36 to -0.66)	Moderate (Jadad score)
Frequency of NEC	4 (393)	RR 0.87 (0.19 to 3.98)	Moderate (Jadad score)
Incidence of late onset sepsis	3 (290)	RR 0.43 (0.30 to 0.61)	Moderate (Jadad score)
Duration of hospital stay	4 (393)	MD -1.35 days (-2.57 to -0.13)	Moderate (Jadad score)
**2. What to feed**			
Brown 2016: Breast milk fortified with carbohydrate, fat and protein vs unfortified breast milk			

Confidential: For Review Only

Weight gain (g/kg/day)	5 (269)	MD 2.82 (1.83 to 3.80)	Low
Gain in length (cm/wk)	3 (189)	MD 0.21 (0.14 to 0.28)	Low
Gain in HC (cm/wk)	3 (189)	MD 0.11 (0.05 to 0.17)	Moderate
Frequency of NEC	7 (539)	RR 1.19 (0.49 to 2.88)	Low
Feed intolerance	5 (255)	RR 0.90 (0.54 to 1.49)	Low
Duration of hospital stay in weeks	2 (210)	MD 0.38 (-0.16 to 0.93)	Low
Amissah 2018a: Breast milk supplemented with fat vs unsupplemented breast milk			
Weight gain (g/kg/day)	1 (14)	MD 0.60 (-2.4 to 3.6)	Very low
Length gain (cm/wk)	1 (14)	MD 0.10 (-0.08 to 0.3)	Very low
Head circumference gain (cm/wk)	1 (14)	MD 0.2 (-0.07 to 0.4)	Very low
Feed intolerance	1 (16)	RR 3.0 (0.1 to 64.3)	Very low
Amissah 2018b: Breast milk supplemented with prebiotic carbohydrate vs unsupplemented breast milk			
Weight gain at age 30 days (g)	1 (75)	MD 160.4 (12.4 to 308.4)	Very low
Frequency of NEC	1 (75)	RR 0.2 (0.02 to 1.3)	Very low
Duration of hospital stay in days	1 (75)	Median (range) supplement vs control: 16 (9-45) vs 25 (11-80) p=0.004	Very low
**3. How to feed**			
Watson 2013: Nasal vs oral placement of feeding tube			
Time to regain birth weight in days	1 (46)	MD 0.90 (-1.27. to 3.07)	NR "Study underpowered to exclude modest but plausible effect sizes"
Time to full feeds (days)	1 (46)	MD -2.7 (-11.9 to 6.5)	NR
Premji 2011: Continuous nasogastric milk vs intermittent bolus feeds			
Weight gain (g/wk)	2 (106)	MD 6.27 (-1.28 to 13.81)	NR
Time to regain birth weight in days	3 (206)	MD -0.31 (-1.65 to 1.03)	NR
Time to full feeds in days	4 (229)	MD 1.82 (-0.44 to 4.08)	NR
Gain in length (cm/wk)	3 (159)	MD 0.07 (-0.04 to 0.18)	NR
Gain in HC (cm/wk)	2 (77)	MD -0.01 (-0.12 to 0.13)	NR
Frequency of proven NEC	4 (270)	RR 2.23 (0.58 to 8.57)	NR

https://mc.manuscriptcentral.com/bmjpo
Confidential: For Review Only

Duration of hospital stay in days	1 (82)	MD -1.00 (-8.62 to 6.62)	NR

Notes: MD = mean difference; CI = confidence interval; NR = not reported; NEC = necrotising enterocolitis

Confidential: For Review Only

When to start feeds

We did not find any reviews comparing the effects of starting feeds before or after 24 hours of life.

How to advance enteral feeds

One review which assessed increasing feeds up to 300 mL/kg/day versus 200 mL/kg/day included one study which recruited 64 babies. Weight gain was greater in the high-volume group with no increase in feeding intolerance, NEC or mortality. The authors assessed the quality of evidence as low to very low because of heterogeneity and risk of bias due to lack of blinding.[22]

One review assessed daily increments of less than 24 mL/kg compared with 30 to 40 mL/kg. There were 10 studies with a total of 3,753 participants. Rate of feed advancement was not associated with weight gain, frequency of NEC or mortality. Slow advancement of feeds may have resulted in a longer time to achieve full feeds, longer hospital stay and a higher risk of sepsis. The authors downgraded the quality of evidence to moderate because there was a lack of blinding in most studies.[20]

One review which assessed advancing feeds to 80ml/kg by the end of the first day compared with 20-30ml/kg included 4 randomised trials and a total of 393 babies.[21] In clinically stable babies, those who had higher volume feeds had lower rates of late-onset sepsis and shorter time to full enteral feeds; these effects were statistically significant in meta-analysis and there was no significant heterogeneity. Higher volume feeds also reduced time to regain birth weight but with significant heterogeneity between the trials. Only two trials reported duration of hospital stay and these trials had significant heterogeneity. The quality of the evidence from the trials was rated as 2 to 3 (low to adequate) on the Jadad scale.

Confidential: For Review Only

What to feed when mother's milk is insufficient or not available

There were no reviews comparing the effect of preterm versus standard term formula.

One review which assessed the effect of fortification of human milk with both energy and protein included 14 trials (13 RCTs and one quasi-RCT) and a total of 1071 participants.[18] Meta-analysis showed a higher weight, length and head circumference at discharge, 12 and 18 months in the fortified group. There was no significant effect on neurodevelopment, necrotising enterocolitis (NEC), feed intolerance, or length of hospital stay. The review authors downgraded the quality of evidence to low as there was significant heterogeneity possibly due to the use of different feeds: some studies used mother's milk only, some mother's milk and donor milk (when mother's milk was insufficient) and others mother's milk and formula.

A systematic review assessed the effect of addition of fat to human milk feeds. The review included one study with only 14 babies; there was no evidence that the addition of fat improved in-hospital growth or affected tolerance of feeds.[23]

A review that assessed the effect of supplementation of human milk with short-chain galacto-oligosaccharides/long-chain fructo-oligosaccharides included one study with 75 participants. There was a significant increase in weight in the supplemented group and decrease in the length of hospital stay but no effect on the incidence of NEC or sepsis.[24] The review authors downgraded the quality of evidence to very low because of a significant risk of bias and the uncertainty resulting from the small sample size.

How to feed

Nasogastric versus orogastric tube versus cup and spoon feeding

Confidential: For Review Only

A review that assessed nasal versus oral placement of enteral feeding tubes and included 1 RCTs with 46 infants found insufficient evidence that the placement of enteral feeding tubes affected time to full enteral feeds, growth and feed intolerance. The review authors assessed the quality of evidence as low due to lack of blinding and failure to report all important outcomes.[25] There was no review assessing cup and spoon feeding in VLBW babies.

Continuous versus bolus feeds

One review which assessed continuous nasogastric versus intermittent bolus feeds included 7 trials and 511babies. There were no significant differences in growth, length of hospital stay or incidence of NEC. The review authors concluded that the benefits and risks of continuous and intermittent feeding could not be assessed from current RCTs and called for future studies to be conducted with consistent feeding protocols and definitions of feed intolerance.[19]

Gravity versus push feeds

We found no reviews assessing gravity versus push feeds.

Methodological quality of the included reviews

We have reported the AMSTAR2 assessments in Table 4.

Confidential: For Review Only

Table 4: AMSTAR2 ratings of included systematic reviews

Review	AMSTAR2 rating																Confidence in findings of review
	Q1	Q2	Q3	Q4	Q5	Q6	Q7	Q8	Q9	Q10	Q11	Q12	Q13	Q14	Q15	Q16	
Abiramalatha 2017	Y	N	N	Y	Y	Y	Y	Y	Y	N	NA	NA	Y	Y	NA	Y	Moderate
Alshaikh 2019	Y	N	N	Y	Y	Y	N	Y	Y	N	Y	N	N	N	Y	Y	Critically low
Amissah 2018a	Y	Y	N	Y	Y	Y	Y	Y	Y	N	NA	NA	Y	Y	NA	N	Moderate
Amissah 2018b	Y	Y	N	Y	Y	Y	Y	Y	Y	Y	NA	NA	Y	Y	NA	Y	High
Brown 2016	Y	N	N	Y	Y	Y	Y	Y	Y	N	Y	Y	Y	Y	N	Y	Moderate
Oddie 2017	Y	N	N	Y	Y	Y	Y	Y	Y	N	Y	Y	Y	Y	Y	Y	Moderate
Premji 2011	Y	PY	N	Y	Y	Y	Y	PY	Y	N	Y	Y	Y	Y	N	Y	Moderate
Watson 2013	Y	N	N	Y	Y	Y	Y	Y	Y	N	NA	NA	Y	Y	NA	Y	Moderate

Notes: AMSTAR2 ratings

1. Did the research questions and inclusion criteria for the review include the components of PICO?
2. Did the report of the review contain an explicit statement that the review methods were established prior to the conduct of the review and did the report justify any significant deviations from the protocol?
3. Did the review authors explain their selection of the study designs for inclusion in the review?
4. Did the review authors use a comprehensive literature search strategy?
5. Did the review authors perform study selection in duplicate?
6. Did the review authors perform data extraction in duplicate?
7. Did the review authors provide a list of excluded studies and justify the exclusions?
8. Did the review authors describe the included studies in adequate detail?
9. Did the review authors use a satisfactory technique for assessing the risk of bias (RoB) in individual studies that were included in the review?
10. Did the review authors report on the sources of funding for the studies included in the review?
11. If meta-analysis was performed, did the review authors use appropriate methods for statistical combination of results?
12. If meta-analysis was performed, did the review authors assess the potential impact of RoB in individual studies on the results of the meta-analysis or other evidence synthesis?
13. Did the review authors account for RoB in primary studies when interpreting/discussing the results of the review?
14. Did the review authors provide a satisfactory explanation for, and discussion of, any heterogeneity observed in the results of the review?

Confidential: For Review Only

15. If they performed quantitative synthesis did the review authors carry out an adequate investigation of publication bias (small study bias) and discuss its likely impact on the results of the review?
16. Did the review authors report any potential sources of conflict of interest, including any funding they received for conducting the review?

Y= Yes; PY=Partial yes; N=No; NA=Not applicable

Confidential: For Review Only

PICO components regarding the research questions and inclusion criteria (Q1) were stated in all reviews. Only 2 reviews stated clearly that the review methods had been established prior to the conduct of the review and justified any significant deviations in methodology where appropriate.[23 24] Although standard Cochrane methodology includes publication of a protocol (Q2) and assessment of the likelihood and impact of publication bias (Q15), we downgraded our quality assessment for Cochrane reviews if these elements were not reported. Methods for searching the literature (Q4), selecting studies for inclusion (Q5), extracting data (Q6) and assessing risk of bias (Q9) were done well in all studies. Only one study reported on the sources of funding for the studies (Q10).[24] All reviews except one[23] reported potential sources of conflict of interest. The only review that was not a Cochrane review had several methodological flaws which led to a critically low level of confidence in its findings.[21] Overall, our confidence in the findings of the reviews was critically low in 1, moderate in 6 and high in only 1.

Discussion

This overview demonstrates that there is a severe lack of evidence to inform basic feeding practices in this highly vulnerable group of infants in low-resource settings in sSA. We found evidence supporting starting feeds at a higher volume, faster advancement of feeds, fortification of breast milk with energy and protein or prebiotics. However, we found only 8 systematic reviews which included small numbers of trials, some of which had few participants. Quality of evidence as assessed by the review authors ranged from very low to moderate. An inherent difficulty in evaluating some feeding approaches is blinding of trial arms. Furthermore, our confidence in the findings was high for only one review. No reviews

Confidential: For Review Only

of RCTs in VLBW babies assessed important issues such as the use of preterm formula, cup and spoon feeding and gravity versus push feeds. Finally, none of the reviews included trials done in the low-income countries of sub-Saharan Africa.

The strengths of this review are that it collates the evidence of randomized controlled trials of feeding interventions for highly vulnerable newborns that are feasible in low-resource settings. It appraises the quality of reviews using a validated measure (AMSTAR2) and updates and expands on the findings of the evidence review commissioned by the World Health Organization published in 2011.[7] A weakness in our review of the evidence that informs feeding practices is that we limited our search to systematic reviews and have not considered other forms of evidence such as RCTs not included in reviews.

Low income countries lack resources to provide parenteral nutrition to VLBW/very preterm babies, particularly in the public health facilities. Hence, the focus is on achieving full enteral feeds as soon as possible, with the potential benefits of reducing length of hospital stay and exposure to hospital-acquired infection, as well as enhancing brain growth. The fetus swallows up to 250mL/kg/day of amniotic fluid which contains trophic factors and provides 10% of daily nutrient requirements suggesting that the newborn gut may tolerate higher fluid volumes than have been previously given.[26] Faster rates of feed advancement (30-40mL/kg/day),[20] and higher volumes of feeds (up to 300mL/kg/day)[22] had some evidence of benefit in terms of higher weight gain, shorter length of hospital stay,[20] shorter time to full enteral feeds[20] and reduced incidence of late-onset sepsis[20] without increasing the risk of NEC. Further trials of these approaches in low income settings are warranted.

Confidential: For Review Only

Multi-nutrient and prebiotic fortification of breast milk shows significant short-term effects but, apart from limited evidence of a lack of effect on psychomotor development at 18 months, evidence on longer-term effect is lacking. Fortifying breast milk with fat alone was not shown to confer any benefit but these results were from a small study and, therefore, the evidence was limited. Fortifying feeds with fat may improve longer term outcomes, given the theoretical benefit of fats on cognition and neurodevelopment.[27] Larger trials in LMIC settings, designed and powered to measure the long-term effects of breast milk fortifiers on growth and development, the incidence of adverse events as well as cost effectiveness are desirable. However, research results may not be applicable until multi-nutrient fortifiers become readily available and affordable in LMICs. A pragmatic alternative would be to test the effects of term formula milk powder for supplementation of calories and macronutrients with due attention to possible adverse effects.

Conclusion

Evidence that informs basic feeding practices of VLBW/very preterm infants in low-resource settings in sSA is poor. There is an urgent need for trials that evaluate pragmatic, inexpensive, low technology and sustainable feeding interventions. Follow-up is needed to include longer-term growth and neurodevelopmental outcomes.

Acknowledgements:

Dr. Symon Kariuki and Kenya Medical Research Institute (KEMRI) librarian Alex Maina assisted with the search and obtaining full texts of reviews. This paper is published with the permission of the Director of the KEMRI-Wellcome Trust Research Programme (Kilifi Centre).

Confidential: For Review Only

Declaration of interest: No conflicts of interest to declare.

Sources of support/funding: This overview is supported by an MRC Confidence in Global Nutrition and Health Research Initiative grant: Improving the survival, growth and development of low birth weight newborns through better nutrition (Reference: MC_PC_MR/R019789/1). The funders had no role in the design or execution of the study, or preparation of the manuscript.

Contributors: AA conceived the study, undertook the literature search, selected reviews, extracted data and wrote the first draft of the manuscript. AT conceived the study, undertook the literature search, selected reviews, checked extracted data and wrote the first draft of the manuscript. SJA conceived the study, arbitrated on selection of reviews, revised the manuscript. IS advised on design of the study, revised the manuscript. NDE advised on design of the study, revised the manuscript All authors read and approved the final manuscript.

Exclusive licence: I, the Submitting Author has the right to grant and does grant on behalf of all authors of the Work (as defined in the below author licence), an exclusive licence and/or a non-exclusive licence for contributions from authors who are: i) UK Crown employees; ii) where BMJ has agreed a CC-BY licence shall apply, and/or iii) in accordance with the terms applicable for US Federal Government officers or employees acting as part of their official duties; on a worldwide, perpetual, irrevocable, royalty-free basis to BMJ Publishing Group Ltd ("BMJ") its licensees and where the relevant Journal is co-owned by BMJ to the co-owners of the Journal, to publish the Work in BMJ Paediatrics Open and any other BMJ products and to exploit all rights, as set out in our licence.

Confidential: For Review Only

The Submitting Author accepts and understands that any supply made under these terms is made by BMJ to the Submitting Author unless you are acting as an employee on behalf of your employer or a postgraduate student of an affiliated institution which is paying any applicable article publishing charge ("APC") for Open Access articles. Where the Submitting Author wishes to make the Work available on an Open Access basis (and intends to pay the relevant APC), the terms of reuse of such Open Access shall be governed by a Creative Commons licence – details of these licences and which Creative Commons licence will apply to this Work are set out in our licence referred to above.

Confidential: For Review Only

References

1. Blencowe H, Cousens S, Oestergaard MZ, et al. National, regional, and worldwide estimates of preterm birth rates in the year 2010 with time trends since 1990 for selected countries: a systematic analysis and implications. *Lancet* 2012;379(9832):2162-72. doi: 10.1016/S0140-6736(12)60820-4 [published Online First: 2012/06/12]

2. Grandi C, Gonzalez A, Zubizarreta J, et al. Perinatal factors associated with neonatal mortality in very low birth weight infants: a multicenter study. *Arch Argent Pediatr* 2016;114(5):426-33. doi: 10.5546/aap.2016.eng.426

10.5546/aap.2016.426 [published Online First: 2016/09/09]

3. Latal-Hajnal B, von Siebenthal K, Kovari H, et al. Postnatal growth in VLBW infants: significant association with neurodevelopmental outcome. *J Pediatr* 2003;143(2):163-70. doi: 10.1067/S0022-3476(03)00243-9 [published Online First: 2003/09/13]

4. Abitbol CL, Moxey-Mims M. Chronic kidney disease: Low birth weight and the global burden of kidney disease. *Nat Rev Nephrol* 2016;12(4):199-200. doi: 10.1038/nrneph.2016.19 [published Online First: 2016/02/24]

5. O'Leary M, Edmond K, Floyd S, et al. A cohort study of low birth weight and health outcomes in the first year of life, Ghana. *Bull World Health Organ* 2017;95(8):574-83. doi: 10.2471/BLT.16.180273 [published Online First: 2017/08/15]

6. Lee AC, Panchal P, Folger L, et al. Diagnostic Accuracy of Neonatal Assessment for Gestational Age Determination: A Systematic Review. *Pediatrics*

Confidential: For Review Only

2017;140(6) doi: 10.1542/peds.2017-1423 [published Online First: 2017/11/19]

7. Organization WH. Guidelines on optimal feeding of low birth weight babies in low and middle income countries. Geneva: WHO 2011.

8. Mosley WH, Chen LC. An analytical framework for the study of child survival in developing countries. 1984. *Bull World Health Organ* 2003;81(2):140-5. [published Online First: 2003/05/22]

9. Nyamtema AS, Urassa DP, van Roosmalen J. Maternal health interventions in resource limited countries: a systematic review of packages, impacts and factors for change. *BMC Pregnancy Childbirth* 2011;11:30. doi: 10.1186/1471-2393-11-30 [published Online First: 2011/04/19]

10. Bradley S, McAuliffe E. Mid-level providers in emergency obstetric and newborn health care: factors affecting their performance and retention within the Malawian health system. *Hum Resour Health* 2009;7:14. doi: 10.1186/1478-4491-7-14 [published Online First: 2009/02/21]

11. Quigley M, Embleton ND, McGuire W. Formula versus donor breast milk for feeding preterm or low birth weight infants. *Cochrane Database Syst Rev* 2018;6:CD002971. doi: 10.1002/14651858.CD002971.pub4 [published Online First: 2018/06/22]

12. Watson J, McGuire W. Transpyloric versus gastric tube feeding for preterm infants. *Cochrane Database Syst Rev* 2013(2):CD003487. doi: 10.1002/14651858.CD003487.pub3 [published Online First: 2013/03/02]

13. Tan-Dy CR, Ohlsson A. Lactase treated feeds to promote growth and feeding tolerance in preterm infants. *Cochrane Database Syst Rev*

Confidential: For Review Only

2013(3):CD004591. doi: 10.1002/14651858.CD004591.pub3 [published Online First: 2013/04/02]

14. Klenoff-Brumberg HL, Genen LH. High versus low medium chain triglyceride content of formula for promoting short term growth of preterm infants. *Cochrane Database Syst Rev* 2003(1):CD002777. doi: 10.1002/14651858.CD002777 [published Online First: 2003/01/22]

15. Verner A, Craig S, McGuire W. Effect of taurine supplementation on growth and development in preterm or low birth weight infants. *Cochrane Database Syst Rev* 2007(4):CD006072. doi: 10.1002/14651858.CD006072.pub2 [published Online First: 2007/10/19]

16. Shea BJ, Reeves BC, Wells G, et al. AMSTAR 2: a critical appraisal tool for systematic reviews that include randomised or non-randomised studies of healthcare interventions, or both. *BMJ* 2017;358:j4008. doi: 10.1136/bmj.j4008 [published Online First: 2017/09/25]

17. Cochrane Handbook for Systematic Reviews of Interventions Version 5.1.0 [updated March 2011]: The Cochrane Collaboration 2011.

18. Brown JV, Embleton ND, Harding JE, et al. Multi-nutrient fortification of human milk for preterm infants. *Cochrane Database Syst Rev* 2016(5):CD000343. doi: 10.1002/14651858.CD000343.pub3 [published Online First: 2016/05/09]

19. Premji SS, Chessell L. Continuous nasogastric milk feeding versus intermittent bolus milk feeding for premature infants less than 1500 grams. *Cochrane Database Syst Rev* 2011(11):CD001819. doi: 10.1002/14651858.CD001819.pub2 [published Online First: 2011/11/11]

Confidential: For Review Only

20. Oddie SJ, Young L, McGuire W. Slow advancement of enteral feed volumes to prevent necrotising enterocolitis in very low birth weight infants. *Cochrane Database Syst Rev* 2017;8:CD001241. doi: 10.1002/14651858.CD001241.pub7 [published Online First: 2017/08/31]

21. Alshaikh B, Dharel D, Yusuf K, et al. Early total enteral feeding in stable preterm infants: a systematic review and meta-analysis. *J Matern Fetal Neonatal Med* 2019:1-8. doi: 10.1080/14767058.2019.1637848 [published Online First: 2019/06/30]

22. Abiramalatha T, Thomas N, Gupta V, et al. High versus standard volume enteral feeds to promote growth in preterm or low birth weight infants. *Cochrane Database Syst Rev* 2017;9:CD012413. doi: 10.1002/14651858.CD012413.pub2 [published Online First: 2017/09/13]

23. Amissah EA, Brown J, Harding JE. Fat supplementation of human milk for promoting growth in preterm infants. *Cochrane Database Syst Rev* 2018;6:CD000341. doi: 10.1002/14651858.CD000341.pub2 [published Online First: 2018/06/21]

24. Amissah EA, Brown J, Harding JE. Carbohydrate supplementation of human milk to promote growth in preterm infants. *Cochrane Database Syst Rev* 2018;8:CD000280. doi: 10.1002/14651858.CD000280.pub2 [published Online First: 2018/08/24]

25. Watson J, McGuire W. Nasal versus oral route for placing feeding tubes in preterm or low birth weight infants. *Cochrane Database Syst Rev* 2013(2):CD003952. doi: 10.1002/14651858.CD003952.pub3 [published Online First: 2013/03/02]

Confidential: For Review Only

26. Mulvihill SJ, Stone MM, Debas HT, et al. The role of amniotic fluid in fetal nutrition. *J Pediatr Surg* 1985;20(6):668-72. doi: 10.1016/s0022-3468(85)80021-x [published Online First: 1985/12/01]

27. Delplanque B, Gibson R, Koletzko B, et al. Lipid Quality in Infant Nutrition: Current Knowledge and Future Opportunities. *J Pediatr Gastroenterol Nutr* 2015;61(1):8-17. doi: 10.1097/MPG.0000000000000818 [published Online First: 2015/04/18]

Confidential: For Review Only

Figure 1 Flow diagram of selection of reviews

Confidential: For Review Only

Figure 1 Flow diagram of selection of reviews

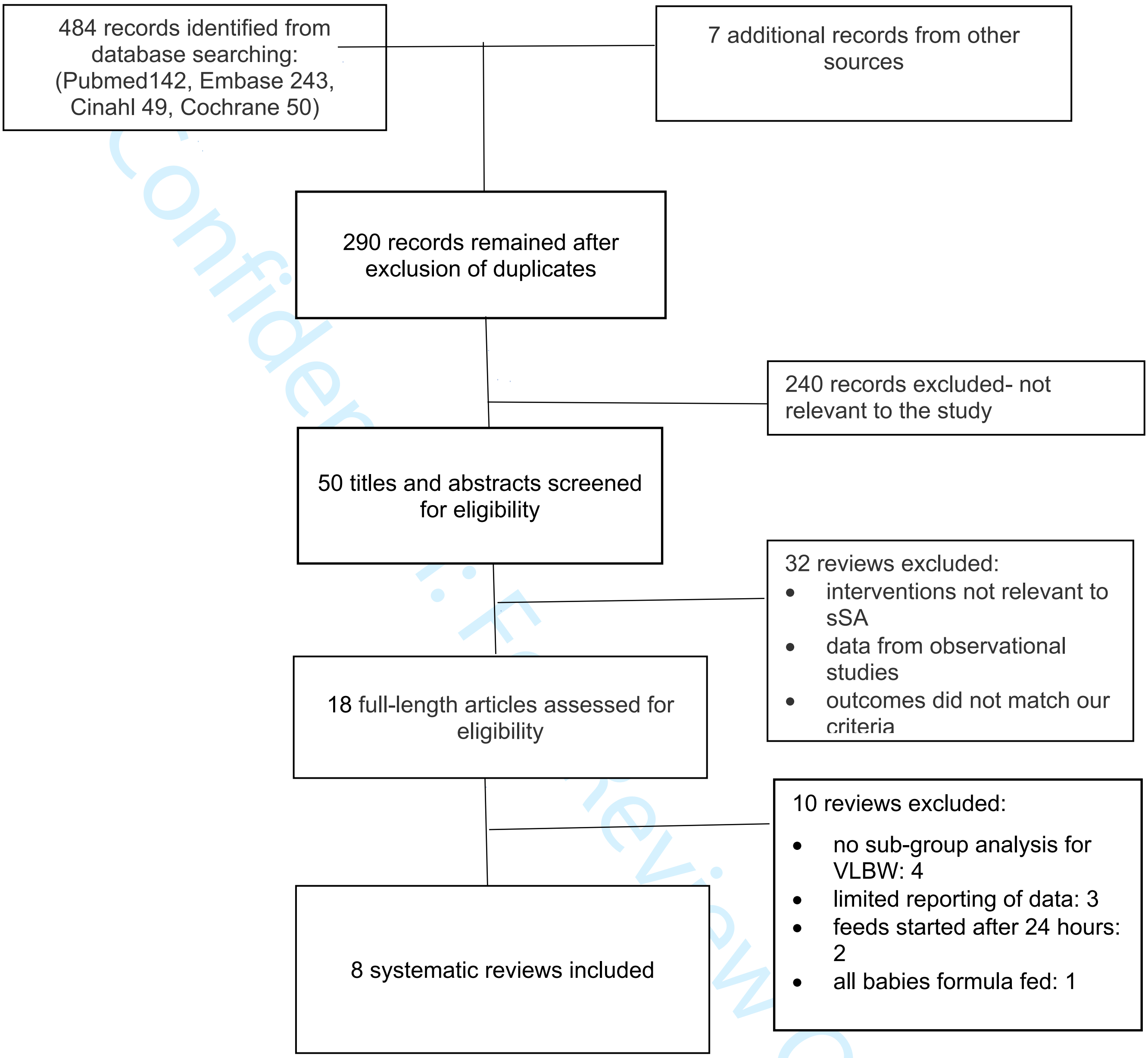

Confidential: For Review Only

Characteristics of Excluded Reviews

Review	Reason for exclusion
Flint 2016[1]	Included term babies. No sub-group analysis for VLBW infants
Morgan 2013[2]	Trophic feeds did not start before 24 hours
Morgan 2014[3]	Enteral feeds did not start before 24 hours
Richards 2014[4]	No RCTs on VLBW infants
Dawson 2012[5]	No sub-group analysis for VLBW infants
Moon 2016[6]	All babies formula fed
Kwok 2019[7]	Limited reporting of growth data
Civardi 2015[8]	Meeting abstract- limited reporting of data
Amissah 2018[9]	No sub-group analysis for VLBW infants
De Pipaon 2019[10]	Meeting abstract – limited reporting of data

References

1. Flint A, New K, Davies MW. Cup feeding versus other forms of supplemental enteral feeding for newborn infants unable to fully breastfeed. *Cochrane Database Syst Rev* 2016(8):CD005092. doi: 10.1002/14651858.CD005092.pub3 [published Online First: 2016/09/01]

2. Morgan J, Bombell S, McGuire W. Early trophic feeding versus enteral fasting for very preterm or very low birth weight infants. *Cochrane Database Syst Rev* 2013(3):CD000504. doi: 10.1002/14651858.CD000504.pub4 [published Online First: 2013/04/02]

Confidential: For Review Only

3. Morgan J, Young L, McGuire W. Delayed introduction of progressive enteral feeds to prevent necrotising enterocolitis in very low birth weight infants. *Cochrane Database Syst Rev* 2014(12):CD001970. doi: 10.1002/14651858.CD001970.pub5 [published Online First: 2014/12/02]

4. Richards R, Foster JP, Psaila K. Continuous versus bolus intragastric tube feeding for preterm and low birth weight infants with gastro-oesophageal reflux disease. *Cochrane Database Syst Rev* 2014(7):CD009719. doi: 10.1002/14651858.CD009719.pub2 [published Online First: 2014/07/18]

5. Dawson JA, Summan R, Badawi N, et al. Push versus gravity for intermittent bolus gavage tube feeding of premature and low birth weight infants. *Cochrane Database Syst Rev* 2012;11:CD005249. doi: 10.1002/14651858.CD005249.pub2 [published Online First: 2012/11/16]

6. Moon K, Rao SC, Schulzke SM, et al. Longchain polyunsaturated fatty acid supplementation in preterm infants. *Cochrane Database Syst Rev* 2016;12:CD000375. doi: 10.1002/14651858.CD000375.pub5 [published Online First: 2016/12/21]

7. Kwok TC, Dorling J, Gale C. Early enteral feeding in preterm infants. *Semin Perinatol* 2019;43(7):151159. doi: 10.1053/j.semperi.2019.06.007 [published Online First: 2019/08/25]

8. Civardi E. Trophic feeding for very preterm or very low birth weight infants. *Italian Journal of Pediatrics* 2015;41(Suppl 1):A3.

9. Amissah EA, Brown J, Harding JE. Protein supplementation of human milk for promoting growth in preterm infants. *Cochrane Database Syst Rev* 2018;6:CD000433. doi: 10.1002/14651858.CD000433.pub2 [published Online First: 2018/06/23]

10. De Pipaon M.S. LMR, Couce M.L., Sanz-Valero J. Effect of long-chain polyunsaturated fatty acid supplementation of preterm infants on infection. A systematic review and meta-analysis. *J Pediatr Gastroenterol Nutr* 2019;68(Supplement 1):1005-06.

Confidential: For Review Only

BMJ Paediatrics Open

Evidence that informs feeding practices in very low birth weight and very preterm infants in sub-Saharan Africa: An overview of systematic reviews

Journal:	*BMJ Paediatrics Open*
Manuscript ID	bmjpo-2020-000724.R1
Article Type:	Original research
Date Submitted by the Author:	19-Jun-2020
Complete List of Authors:	Akindolire, Abimbole; University of Ibadan College of Medicine<br>Talbert, Alison; Centre for Geographic Medicine Research Coast<br>Sinha, Ian; Alder Hey Children's NHS Foundation Trust, Respiratory Medicine<br>Embleton, Nick; Newcastle University<br>Allen, Stephen; Liverpool School of Tropical Medicine
Keywords:	Neonatology

SCHOLARONE™
Manuscripts

BMJ

I, the Submitting Author has the right to grant and does grant on behalf of all authors of the Work (as defined in the below author licence), an exclusive licence and/or a non-exclusive licence for contributions from authors who are: i) UK Crown employees; ii) where BMJ has agreed a CC-BY licence shall apply, and/or iii) in accordance with the terms applicable for US Federal Government officers or employees acting as part of their official duties; on a worldwide, perpetual, irrevocable, royalty-free basis to BMJ Publishing Group Ltd ("BMJ") its licensees and where the relevant Journal is co-owned by BMJ to the co-owners of the Journal, to publish the Work in this journal and any other BMJ products and to exploit all rights, as set out in our licence.

The Submitting Author accepts and understands that any supply made under these terms is made by BMJ to the Submitting Author unless you are acting as an employee on behalf of your employer or a postgraduate student of an affiliated institution which is paying any applicable article publishing charge ("APC") for Open Access articles. Where the Submitting Author wishes to make the Work available on an Open Access basis (and intends to pay the relevant APC), the terms of reuse of such Open Access shall be governed by a Creative Commons licence – details of these licences and which Creative Commons licence will apply to this Work are set out in our licence referred to above.

Other than as permitted in any relevant BMJ Author's Self Archiving Policies, I confirm this Work has not been accepted for publication elsewhere, is not being considered for publication elsewhere and does not duplicate material already published. I confirm all authors consent to publication of this Work and authorise the granting of this licence.

Confidential: For Review Only

Review title

Evidence that informs feeding practices in very low birthweight and very preterm infants in sub-Saharan Africa: An overview of systematic reviews

Abimbola E Akindolire MB BS,[1*] Alison W Talbert MRCP,[2*] Ian Sinha PhD,[3] Nicholas D Embleton MD,[4] Stephen J Allen MD [3,5] on behalf of the Neonatal Nutrition Network (NeoNuNet)

*Joint first authors

Corresponding author:

Dr. Alison Talbert

KEMRI-Wellcome Trust Research Programme

P.O. Box 230

Kilifi 80108

Kenya

Phone: +254 729 218993

Email: atalbert@kemri-wellcome.org

Affiliations

1. University College Hospital, Ibadan, Nigeria
2. KEMRI-Wellcome Trust Research Programme, Kilifi, Kenya
3. Alder Hey Children's Hospital NHS Trust, Liverpool, UK
4. Newcastle University, Newcastle, UK
5. Liverpool School of Tropical Medicine, Liverpool, UK

Confidential: For Review Only

Neonatal Nutrition Network members: Olusegun Akinyinka (College of Medicine, University of Ibadan, Nigeria); Dominic D Omuru (Maitama District Hospital, Abuja, Nigeria); Chinyere Ezeaka (Lagos University Teaching Hospital, Nigeria); Beatrice N Ezenwa (Lagos University Teaching Hospital, Nigeria); Iretiola B Fajolu (Lagos University Teaching Hospital, Nigeria); Zainab O Imam (Massey St. Children's Hospital, Lagos, Nigeria); Martha K Mwangome (KEMRI Wellcome Trust Research Programme, Kilifi, Kenya); Alison W Talbert (KEMRI Wellcome Trust Research Programme, Kilifi, Kenya); Pauline EA Andang'o (Maseno University, Kenya); Walter Otieno (Jaramogi Oginga Odinga Teaching and Referral Hospital, Kisumu, Kenya & Maseno University, Kenya); Grace M Nalwa (Jaramogi Oginga Odinga Teaching and Referral Hospital, Kisumu, Kenya & Maseno University, Kenya); Ian Sinha (Alder Hey Children's Hospital, Liverpool, UK); Janneke van de Wijgert (University of Liverpool, Liverpool, UK); Melissa Gladstone (University of Liverpool, Liverpool, UK); Kevin Mortimer (Liverpool School of Tropical Medicine, Liverpool, UK); Graham Devereux (Liverpool School of Tropical Medicine, Liverpool, UK); Ismaela Abubakar (Liverpool School of Tropical Medicine, Liverpool, UK); Stephen Turner (University of Aberdeen, Aberdeen, UK); Nicholas Embleton (Newcastle University, Newcastle, UK); Helen M Nabwera (Liverpool School of Tropical Medicine, Liverpool, UK); Stephen J Allen (Liverpool School of Tropical Medicine, Liverpool, UK); Isa Abdulkadir (Ahmadu Bello University, Zaria, Nigeria); Olukemi O Tongo (College of Medicine, University of Ibadan, Nigeria); Abimbola E Akindolire (College of Medicine, University of Ibadan, Nigeria).

Confidential: For Review Only

Number of figures 1

Number of tables 4

Number of words 4404

Number of references 32

Number of black and white figures 1

Number of coloured figures 0

Number of supplemental files 1

Confidential: For Review Only

Abstract

Background Optimal feeding of very low birthweight (VLBW <1500g)/very preterm (gestation <32 weeks) infants in resource-limited settings in sub-Saharan Africa (sSA) is critical to reducing high mortality and poor outcomes.

Objective To review evidence on feeding of VLBW/very preterm infants relevant to sSA.

Methods We searched the Cochrane Database of Systematic Reviews, EMBASE, PUBMED and CINAHL from inception to July 2019 to identify reviews of randomised and quasi-randomised controlled trials of feeding VLBW/very preterm infants. We focused on interventions that are readily available in sSA. Primary outcomes were weight gain during hospital stay and time to achieve full enteral feeds (120 mls/kg/day). Secondary outcomes were growth, common morbidities, mortality, duration of hospital stay and cognitive development. Quality of evidence (QOE) was assessed using the Measurement Tool to Assess Systematic Reviews (AMSTAR2).

Results Eight systematic reviews were included. Higher feed volume of day 1 (80 ml/kg) reduced late-onset sepsis and time to full enteral feeds, and higher feed volume (up to 300 mls/kg/day) improved weight gain without adverse events (QOE: low-moderate). Rapid advancement of feeds (30-40 ml/kg/day) was not associated with harm. Breast milk fortification with energy and protein increased growth and with prebiotics increased growth and reduced duration of admission (QOE: low -very low) and did not result in harm. Evidence regarding feeding tube placement and continuous versus bolus feeds was insufficient to draw conclusions. We found no reviews meeting our selection criteria regarding when to start feeds, use of preterm

Confidential: For Review Only

formula, cup-and-spoon feeding or gravity versus push feeds and none of the reviews included trials from low-income countries of sSA.

Conclusions The evidence base informing feeding of VLBW/very preterm babies in resource-limited settings in sSA is extremely limited. Pragmatic studies are needed to generate evidence to guide management and improve outcomes for these highly vulnerable infants.

Protocol registration

http://www.crd.york.ac.uk/PROSPERO/display_record.php?ID=CRD42019140204

Abstract 300 words

For Review Only

What is known already on this topic?

- Although survival is improving, mortality and outcomes for VLBW/very preterm infants in resource-limited settings in sSA remain poor.
- Optimising feeding is critical to improving survival, healthy growth and development and reducing morbidity.
- International and national protocols inform the feeding of VLBW/very preterm infants but evidence relevant to resource-limited settings is limited.

What this study adds

- No trials of pragmatic feeding interventions have been conducted in resource-limited settings in sSA.
- Higher feed volumes, rapid advancement of feeds and fortification of breast milk may be beneficial in achieving full feeds, improving growth and reducing morbidity without adverse effects.
- Trials of pragmatic feeding protocols in resource-limited settings are needed urgently to better inform clinical practice.

For peer review only

Background

Worldwide about 15 million babies are born preterm annually and it is estimated that 28% of these births occur in sub-Saharan Africa (sSA) [1] An increasing number of very low birth weight (VLBW; BW<1500g) babies are surviving till discharge in low and middle income countries (LMICs).[2] Optimal nutrition is key for survival, prevention of adverse events such as sepsis and reducing the length of the hospital stay.[3] In addition, there is strong evidence that early nutrition impacts on multi-organ developmental patterning, immune, cardiac and respiratory function and longer term cognitive outcome as well as a range of chronic conditions such as diabetes and cardiovascular disease in adulthood which place a substantial burden on healthcare systems.[3-7]

Most very preterm infants (gestation <32 weeks) have yet to develop coordinated oromotor skills of sucking, swallowing and breathing that allow safe breastfeeding and, therefore, other methods of feeding are necessary. Uncertainty about gestational age due to a lack of reliable first trimester ultrasound scan is common in LMICs and birthweight is often used to identify infants who are preterm, growth-retarded or both.[8] In most neonatal units (NNUs) in LMICs, parenteral nutrition is not available.[9] Trials on enteral feeding interventions have been done mainly in high and middle-income countries. The generalisability of their findings to feeding VLBW infants in most of sSA is difficult to assess due to multiple differences in maternal, pregnancy, delivery, neonatal and environmental factors and also the levels of care and clinical monitoring provided in high versus low-middle income countries.[10-12]

Although internationally agreed guidelines on optimal feeding of low birthweight infants in LMICs, including some recommendations for VLBW infants, were published by the

Confidential: For Review Only

World Health Organization in 2011, these lacked a strong evidence-base especially for LMIC settings.[9] This led us to search the literature for evidence from reviews of studies conducted in, or applicable to, sub-Saharan Africa. The aim of our study was to review the evidence for pragmatic, low cost interventions to optimise the feeding of VLBW/very preterm infants in low-resource settings in sSA. We systematically searched for evidence on when to start enteral feeds; how to advance feeds; what to feed when mother's own milk is insufficient or not available and how to feed.

Methods and analysis

Protocol registration

We registered the overview protocol prospectively on the PROSPERO International Prospective Register of Systematic Reviews (http://www.crd.york.ac.uk/PROSPERO/display_record.php?ID=CRD42019140204)

Patient and public involvement

There was no patient or public involvement in the planning or execution of this overview.

Search strategy

We searched the Cochrane Database of Systematic reviews, EMBASE, PUBMED and CINAHL from inception to July 2019 for systematic reviews and meta-analyses of randomised controlled trials (RCTs) that assessed the key questions listed above irrespective of language of publication. The key search terms were: very low birthweight or preterm AND (enteral feeding or enteral nutrition) AND (systematic review or meta-analysis). We checked the reference lists of the identified articles and published guidelines for systematic reviews not previously identified.

Confidential: For Review Only

Selection of reviews

Table 1: Criteria for inclusion of reviews

Participants<br>• Neonates (babies aged < 28 days)<br>• Birth weight less <1500g and/or gestation <32 weeks
Interventions<br>• When to start enteral feeds:<br>○ Age <24 hours or ≥24 hours<br>• How to advance feeds:<br>○ Slow (15-20 mL/kg/day) vs. fast (30-35 mL/kg/day)<br>• What to feed when mother's milk is insufficient or not available:<br>○ Preterm formula vs. standard infant formula<br>○ Unfortified vs. fortified breastmilk<br>• How to feed:<br>○ Nasogastric vs. orogastric tube vs. cup and spoon feeding<br>○ Continuous vs. bolus feeds<br>○ Gravity vs. push feeds<br>Interventions not currently widely available in sSA were excluded
Outcomes<br>• Primary outcomes<br>○ Weight gain during hospital stay (g/kg/day or time to regain birthweight)<br>○ Time in days to achieve full enteral feeds (120 mL/kg/day)<br>• Secondary outcomes<br>○ Gain in length and head circumference during hospital stay<br>○ Frequency of necrotising enterocolitis, sepsis<br>○ Duration of hospital stay in days<br>○ Cognitive development<br>○ Mortality
Reviews<br>• Included: peer reviewed systematic reviews of randomised clinical trials (RCTs) including cluster randomised or quasi-randomised trials<br>• Excluded: non-randomised designs

Confidential: For Review Only

Criteria for inclusion of reviews are shown in Table 1. Reviews reporting interventions not currently widely available in sSA, such as use of donor breast milk,[13] transpyloric feeding,[14] and specific nutritional supplements[15-17] were excluded. Two authors independently screened titles and abstracts to identify relevant reviews for full-text review. Disagreements were resolved by discussion involving a third author.

Data extraction and management

One author extracted data from all selected reviews into a spreadsheet (Microsoft Excel) including number and settings of the included trials, total number and characteristics of participants, intervention(s) assessed, outcomes measured and major limitations. A second author crosschecked the extracted data for accuracy.

Assessment of methodological quality of included reviews

Two authors independently assessed the quality of each review using the revised AMSTAR 2 (A Measurement Tool to Assess systematic Reviews) tool.[18] Discrepancies were resolved by discussion and, if necessary, arbitration by a third author. The level of confidence in the findings of the reviews was assessed according to the number of critical and minor flaws in the methodology.[18]

Outcomes

Primary outcomes were weight gain during hospital stay and time to achieve full enteral feeds (120 mls/kg/day). These were selected on the basis that they are recorded routinely in LMIC settings and are directly relevant to clinical practice. Secondary outcomes included growth, other important clinical outcomes such as sepsis, necrotising enterocolitis (NEC), death, duration of hospital stay and cognitive development (Table 1).

Data synthesis

We presented the findings for the primary and secondary outcomes for each intervention.[19] We reported other outcome data in a narrative form. The relevance of the findings to feeding VLBW/very preterm infants in sSA was evaluated.

Results

The search yielded 491 reviews (Figure 1). After excluding duplicate and updated reviews, the titles and abstracts of 50 reviews were assessed for inclusion. The full texts of 18 reviews were screened of which 10 were excluded (see Supplementary File 1). Eight reviews met our inclusion criteria (Table 2). The number of studies included in reviews ranged from 1 to 14 and the number of participants from 14 to 3753. Some of the findings from studies were pooled in a meta-analysis in 4 reviews.[20-23] None of the reviews included studies from low income countries including in sSA. Table 3 summarizes the outcomes measured and estimates of size of effects of each intervention.

Confidential: For Review Only

Table 2: Characteristics of included reviews

Review	Population	Intervention arm	Comparison arm	Outcomes reported
**1. How to advance feeds**				
Abiramalatha 2017	Preterm (<37 weeks) or low birth weight (<2500g) infants	High volume enteral feeds up to 300mL/kg/day	Standard volume enteral feeds ≤200mL/kg/day	• Weight gain during hospital stay<br>• Feed intolerance<br>• Necrotizing enterocolitis
Oddie 2017	Enterally fed very preterm (<32 weeks) or VLBW infants	Faster advancement of enteral feeds	Advancement of enteral feeds no faster than 24mL/kg (birth weight or current weight) per day	• Feed intolerance<br>• Time to establish full enteral feeding<br>• Incidence of invasive infections<br>• Necrotizing enterocolitis<br>• Duration of hospital stay<br>• Mortality
Alshaikh 2019	VLBW (1000-<1500g) stable newborn infants	Enteral feeds started on day 1 at 80mL/kg/day and increased by 20mL/kg/day	Enteral feeds started on day 1 at ≤30mL/kg/day and increased by 20mL/kg/day	• Time to regain birth weight<br>• Feed intolerance: time to establish full enteral feeds.<br>• Necrotizing enterocolitis and sepsis<br>• Duration of hospital stay
**2. What to feed**				
Brown 2016	Preterm (<37 weeks) and low birth weight (<2500g) infants receiving breast milk in hospital	Fortification of breast milk with energy (carbohydrate or fat) and protein. Fortifiers could additionally include micronutrients and vitamins	Breast milk not fortified with energy or protein but can receive micronutrients and vitamins	• Growth: weight, length, head circumference<br>• Length of hospital stay<br>• Feed intolerance<br>• Necrotizing enterocolitis<br>• Bone mineralization: serum alkaline phosphatase, bone mineral content<br>• Neurodevelopmental outcomes at 18 months: mental development index and psychomotor development index

Confidential: For Review Only

Amissah 2018a	Preterm (<37 weeks) infants receiving enteral feeding of human milk in hospital	Human milk with additional fat supplementation	Human milk without additional fat supplementation	• Growth: weight, length, head circumference
Amissah 2018b	Preterm (<37 weeks) infants receiving human milk in hospital	Human milk with additional carbohydrate supplementation	Human milk without additional carbohydrate supplementation	• Weight at day 30 of age<br>• Duration of hospital stay<br>• Feed intolerance<br>• Necrotizing enterocolitis
**3. How to feed**				
Watson 2013	Preterm (<37 weeks) or low birth weight (<2500g) infants receiving tube feeding	Nasal placement of feeding tubes	Oral placement of feeding tubes	• Time to establish full tube feeds<br>• Time to regain birth weight<br>• Weight gain<br>• Time to independence from supplemental oxygen
Premji 2011	VLBW infants with no prior history of feeding or feed intolerance and no congenital anomalies that might interfere with establishing enteral feeds	Continuous nasogastric feeding with human milk or infant formula	Intermittent bolus nasogastric feeding with human milk or infant formula	• Time to establish full enteral feeds<br>• Time to establish full oral feeds<br>• Feed intolerance<br>• Days on TPN<br>• Time to regain birth weight<br>• Growth: weight, length, head circumference, triceps skinfold thickness<br>• Duration of hospital stay<br>• Days to discharge weight of 2040g<br>• Days on mechanical ventilation<br>• Proven or probable necrotizing enterocolitis<br>• Failure to complete protocol due to feed intolerance<br>• Apnoea

Notes: VLBW = very low birthweight (<1500g); TPN = total parenteral nutrition

Confidential: For Review Only

Table 3: Findings from systematic reviews

**Review/Outcome**	**No. of studies (no. of babies)**	**Results** MD or RR (95% CI)	**Quality of evidence** (GRADE assessment)
**1. How to advance feeds**			
Abiramalatha 2017: High volume vs standard volume feeds			
Weight gain (g/kg/day)	1 (64)	MD 6.2 (2.71 to 9.69)	Low
Frequency of NEC	1 (64)	RR 1.03 (0.07 to 15.78)	Very low
Feed intolerance	1 (64)	RR 1.81 (0.89 to 3.67)	Low
Mortality	1 (64)	No death in any group	NR
Oddie 2017: Slow vs faster rate or few advancement			
Weight gain at discharge	1 (2804)	MD 0.0 (-0.08 to 0.08)	NR
Time to full feeds	8 (3551)	Longer time in slow advancement group. CI NR	NR
Gain in HC z score at discharge	1 (2804)	MD 0.0 (-0.13 to 0.13)	NR
Frequency of NEC	10 (3738)	RR 1.07 (CI 0.83 to 1.39)	Moderate
Frequency of sepsis	8 (3391)	RR 1.15 (1.00 to 1.32)	Low
Duration of hospital stay in days		2 trials reported longer duration in slow advancement group	NR
Mortality	9(3553)	RR 1.15 (0.93 to 1.42)	Moderate
Alshaikh 2019: Early total enteral feeds vs early partial enteral feeds			
Time to regain birth weight	2 (149)	MD -1.15 days (-1.86 to -0.45)	Moderate (Jadad score)
Feed intolerance	3 (347)	RR 0.78 (0.38 to 1.59)	Moderate (Jadad score)
Time to full enteral feeds	2 (164)	MD -1.01 days (-1.36 to -0.66)	Moderate (Jadad score)
Frequency of NEC	4 (393)	RR 0.87 (0.19 to 3.98)	Moderate (Jadad score)
Incidence of late onset sepsis	3 (290)	RR 0.43 (0.30 to 0.61)	Moderate (Jadad score)
Duration of hospital stay	4 (393)	MD -1.35 days (-2.57 to -0.13)	Moderate (Jadad score)
**2. What to feed**			
Brown 2016: Breast milk fortified with carbohydrate, fat and protein vs unfortified breast milk			
Weight gain (g/kg/day)	5 (269)	MD 2.82 (1.83 to 3.80)	Low
Gain in length (cm/wk)	3 (189)	MD 0.21 (0.14 to 0.28)	Low

Confidential: For Review Only

Gain in HC (cm/wk)	3 (189)	MD 0.11 (0.05 to 0.17)	Moderate
Frequency of NEC	7 (539)	RR 1.19 (0.49 to 2.88)	Low
Feed intolerance	5 (255)	RR 0.90 (0.54 to 1.49)	Low
Duration of hospital stay in weeks	2 (210)	MD 0.38 (-0.16 to 0.93)	Low
Amissah 2018a: Breast milk supplemented with fat vs unsupplemented breast milk			
Weight gain (g/kg/day)	1 (14)	MD 0.60 (-2.4 to 3.6)	Very low
Length gain (cm/wk)	1 (14)	MD 0.10 (-0.08 to 0.3)	Very low
Head circumference gain (cm/wk)	1 (14)	MD 0.2 (-0.07 to 0.4)	Very low
Feed intolerance	1 (16)	RR 3.0 (0.1 to 64.3)	Very low
Amissah 2018b: Breast milk supplemented with prebiotic carbohydrate vs unsupplemented breast milk			
Weight gain at age 30 days (g)	1 (75)	MD 160.4 (12.4 to 308.4)	Very low
Frequency of NEC	1 (75)	RR 0.2 (0.02 to 1.3)	Very low
Duration of hospital stay in days	1 (75)	Median (range) supplement vs control: 16 (9-45) vs 25 (11-80) p=0.004	Very low
**3. How to feed**			
Watson 2013: Nasal vs oral placement of feeding tube			
Time to regain birth weight in days	1 (46)	MD 0.90 (-1.27. to 3.07)	NR "Study underpowered to exclude modest but plausible effect sizes"
Time to full feeds (days)	1 (46)	MD -2.7 (-11.9 to 6.5)	NR
Premji 2011: Continuous nasogastric milk vs intermittent bolus feeds			
Weight gain (g/wk)	2 (106)	MD 6.27 (-1.28 to 13.81)	NR
Time to regain birth weight in days	3 (206)	MD -0.31 (-1.65 to 1.03)	NR
Time to full feeds in days	4 (229)	MD 1.82 (-0.44 to 4.08)	NR
Gain in length (cm/wk)	3 (159)	MD 0.07 (-0.04 to 0.18)	NR
Gain in HC (cm/wk)	2 (77)	MD -0.01 (-0.12 to 0.13)	NR
Frequency of proven NEC	4 (270)	RR 2.23 (0.58 to 8.57)	NR
Duration of hospital stay in days	1 (82)	MD -1.00 (-8.62 to 6.62)	NR

Notes: MD = mean difference; CI = confidence interval; NR = not reported; NEC = necrotising enterocolitis

Confidential: For Review Only

When to start feeds

We did not find any reviews comparing the effects of starting feeds before or after 24 hours of life.

How to advance enteral feeds

One review which assessed increasing feeds up to 300 mL/kg/day versus 200 mL/kg/day included one study which recruited 64 babies. Weight gain was greater in the high-volume group with no increase in feeding intolerance, NEC or mortality. The authors assessed the quality of evidence as low to very low because of heterogeneity and risk of bias due to lack of blinding.[24]

One review assessed daily increments of less than 24 mL/kg compared with 30 to 40 mL/kg. There were 10 studies with a total of 3,753 participants. Rate of feed advancement was not associated with weight gain, frequency of NEC or mortality. Slow advancement of feeds may have resulted in a longer time to achieve full feeds, longer hospital stay and a higher risk of sepsis. The authors downgraded the quality of evidence to moderate because there was a lack of blinding in most studies.[22]

One review which assessed advancing feeds to 80ml/kg by the end of the first day compared with 20-30ml/kg included 4 randomised trials and a total of 393 babies.[23] In clinically stable babies, those who had higher volume feeds had lower rates of late-onset sepsis and shorter time to full enteral feeds; these effects were statistically significant in meta-analysis and there was no significant heterogeneity. Higher volume feeds also reduced time to regain birth weight but with significant heterogeneity between the trials. Only two trials reported duration of hospital stay and these trials had significant heterogeneity. The quality of the evidence from the trials was rated as 2 to 3 (low to adequate) on the Jadad scale.

Confidential: For Review Only

What to feed when mother's milk is insufficient or not available

There were no reviews comparing the effect of preterm versus standard term formula.

One review which assessed the effect of fortification of human milk with both energy and protein included 14 trials (13 RCTs and one quasi-RCT) and a total of 1071 participants.[20] Meta-analysis showed a higher weight, length and head circumference at discharge, 12 and 18 months in the fortified group. There was no significant effect on neurodevelopment, necrotising enterocolitis (NEC), feed intolerance, or length of hospital stay. The review authors downgraded the quality of evidence to low as there was significant heterogeneity possibly due to the use of different feeds: some studies used mother's milk only, some mother's milk and donor milk (when mother's milk was insufficient) and others mother's milk and formula.

A systematic review assessed the effect of addition of fat to human milk feeds. The review included one study with only 14 babies; there was no evidence that the addition of fat improved in-hospital growth or affected tolerance of feeds.[25] The quality of evidence was very low due to incomplete description of the methodology and small sample size.

A review that assessed the effect of supplementation of human milk with short-chain galacto-oligosaccharides/long-chain fructo-oligosaccharides included one study with 75 participants. There was a significant increase in weight in the supplemented group and decrease in the length of hospital stay but no effect on the incidence of NEC or sepsis.[26] The review authors downgraded the quality of evidence to very low because of a significant risk of bias and the uncertainty resulting from the small sample size.

How to feed

Confidential: For Review Only

Nasogastric versus orogastric tube versus cup and spoon feeding

A review that assessed nasal versus oral placement of enteral feeding tubes and included 1 RCTs with 46 infants found insufficient evidence that the placement of enteral feeding tubes affected time to full enteral feeds, growth and feed intolerance. The review authors assessed the quality of evidence as low due to lack of blinding and failure to report all important outcomes.[27] There was no review assessing cup and spoon feeding in VLBW babies.

Continuous versus bolus feeds

One review which assessed continuous nasogastric versus intermittent bolus feeds included 7 trials and 511babies. There were no significant differences in growth, length of hospital stay or incidence of NEC. The review authors concluded that the benefits and risks of continuous and intermittent feeding could not be assessed from current RCTs and called for future studies to be conducted with consistent feeding protocols and definitions of feed intolerance.[21]

Gravity versus push feeds

We found no reviews assessing gravity versus push feeds.

Methodological quality of the included reviews

We have reported the AMSTAR2 assessments in Table 4.

Confidential: For Review Only

Table 4: AMSTAR2 ratings of included systematic reviews

Review	AMSTAR2 rating																Confidence in findings of review
	Q1	Q2	Q3	Q4	Q5	Q6	Q7	Q8	Q9	Q10	Q11	Q12	Q13	Q14	Q15	Q16	
Abiramalatha 2017	Y	N	N	Y	Y	Y	Y	Y	Y	N	NA	NA	Y	Y	NA	Y	Moderate
Alshaikh 2019	Y	N	N	Y	Y	Y	N	Y	Y	N	Y	N	N	N	Y	Y	Critically low
Amissah 2018a	Y	Y	N	Y	Y	Y	Y	Y	Y	N	NA	NA	Y	Y	NA	N	Moderate
Amissah 2018b	Y	Y	N	Y	Y	Y	Y	Y	Y	Y	NA	NA	Y	Y	NA	Y	High
Brown 2016	Y	N	N	Y	Y	Y	Y	Y	Y	N	Y	Y	Y	Y	N	Y	Moderate
Oddie 2017	Y	N	N	Y	Y	Y	Y	Y	Y	N	Y	Y	Y	Y	Y	Y	Moderate
Premji 2011	Y	PY	N	Y	Y	Y	Y	PY	Y	N	Y	Y	Y	Y	N	Y	Moderate
Watson 2013	Y	N	N	Y	Y	Y	Y	Y	Y	N	NA	NA	Y	Y	NA	Y	Moderate

Notes: AMSTAR2 ratings
1. Did the research questions and inclusion criteria for the review include the components of PICO?
2. Did the report of the review contain an explicit statement that the review methods were established prior to the conduct of the review and did the report justify any significant deviations from the protocol?
3. Did the review authors explain their selection of the study designs for inclusion in the review?
4. Did the review authors use a comprehensive literature search strategy?
5. Did the review authors perform study selection in duplicate?
6. Did the review authors perform data extraction in duplicate?
7. Did the review authors provide a list of excluded studies and justify the exclusions?
8. Did the review authors describe the included studies in adequate detail?
9. Did the review authors use a satisfactory technique for assessing the risk of bias (RoB) in individual studies that were included in the review?
10. Did the review authors report on the sources of funding for the studies included in the review?
11. If meta-analysis was performed, did the review authors use appropriate methods for statistical combination of results?
12. If meta-analysis was performed, did the review authors assess the potential impact of RoB in individual studies on the results of the meta-analysis or other evidence synthesis?
13. Did the review authors account for RoB in primary studies when interpreting/discussing the results of the review?
14. Did the review authors provide a satisfactory explanation for, and discussion of, any heterogeneity observed in the results of the review?

Confidential: For Review Only

15. If they performed quantitative synthesis did the review authors carry out an adequate investigation of publication bias (small study bias) and discuss its likely impact on the results of the review?
16. Did the review authors report any potential sources of conflict of interest, including any funding they received for conducting the review?

Y= Yes; PY=Partial yes; N=No; NA=Not applicable

Confidential: For Review Only

PICO components regarding the research questions and inclusion criteria (Q1) were stated in all reviews. Only 2 reviews stated clearly that the review methods had been established prior to the conduct of the review and justified any significant deviations in methodology where appropriate.[25 26] Although standard Cochrane methodology includes publication of a protocol (Q2) and assessment of the likelihood and impact of publication bias (Q15), we downgraded our quality assessment for Cochrane reviews if these elements were not reported. Methods for searching the literature (Q4), selecting studies for inclusion (Q5), extracting data (Q6) and assessing risk of bias (Q9) were done well in all studies. Only one study reported on the sources of funding for the studies (Q10).[26] All reviews except one[25] reported potential sources of conflict of interest. The only review that was not a Cochrane review had several methodological flaws which led to a critically low level of confidence in its findings.[23] Overall, our confidence in the findings of the reviews was critically low in 1, moderate in 6 and high in only 1.

Discussion

This overview demonstrates that there is a severe lack of evidence to inform basic feeding practices in this highly vulnerable group of infants in low-resource settings in sSA. We found evidence supporting starting feeds at a higher volume, faster advancement of feeds, fortification of breast milk with energy and protein or prebiotics. However, we found only 8 systematic reviews which included small numbers of trials, some of which had few participants. Quality of evidence as assessed by the review authors ranged from very low to moderate. An inherent difficulty in evaluating some feeding approaches is blinding of trial arms. Furthermore, our confidence in the findings was high for only one review. No reviews

Confidential: For Review Only

of RCTs in VLBW babies assessed important issues such as the use of preterm formula, cup and spoon feeding and gravity versus push feeds. Finally, none of the reviews included trials done in the low-income countries of sub-Saharan Africa.

The strengths of this review are that it collates the evidence of randomized controlled trials of feeding interventions for highly vulnerable newborns that are feasible in low-resource settings. It appraises the quality of reviews using a validated measure (AMSTAR2) and updates and expands on the findings of the evidence review commissioned by the World Health Organization published in 2011.[9] A weakness in our review of the evidence that informs feeding practices is that we limited our search to systematic reviews and have not considered other forms of evidence such as RCTs not included in reviews.

Human donor milk has been recommended by the WHO as the best alternative when mother's own milk is not available but there are few human milk banks operating in sSA.[9] The practice of wet-nursing and informal milk sharing has been carried out in many societies in Africa but we did not find any systematic reviews in hospital settings. In recent years these practices have been discouraged due to fears of HIV transmission but qualitative studies in sSA have explored parents' and healthcare workers' attitudes to using screened and pasteurised donor human milk through facility-based human milk banks.[28 29]

Low income countries lack resources to provide parenteral nutrition to VLBW/very preterm babies, particularly in the public health facilities. Hence, the focus is on achieving full enteral feeds as soon as possible, with the potential benefits of reducing

Confidential: For Review Only

length of hospital stay and exposure to hospital-acquired infection, as well as enhancing brain growth. Initiation of enteral feeding was previously delayed through fears of adverse effects on the immature gut, and we did not find any systematic reviews comparing initiation in the first 24 hours with later introduction. A systematic review, that was excluded because the cut off for early versus delayed initiation was 4 days, found no evidence of decreased risk of NEC if feeds were delayed, but did find a delay in achieving full enteral feeds.[30] The definition of full enteral feeds was not consistent within and between reviews with some studies reporting milk volumes and others kilocalories consumed. Some studies specified for how long feeds were tolerated before meeting the definition. This makes direct comparisons of this outcome measure impossible.

The fetus swallows up to 250mL/kg/day of amniotic fluid which contains trophic factors and provides 10% of daily nutrient requirements suggesting that the newborn gut may tolerate higher fluid volumes than have been previously given.[31] Faster rates of feed advancement (30-40mL/kg/day),[22] and higher volumes of feeds (up to 300mL/kg/day)[24] had some evidence of benefit in terms of higher weight gain, shorter length of hospital stay,[22] shorter time to full enteral feeds[22] and reduced incidence of late-onset sepsis[22] without increasing the risk of NEC. Further trials of these approaches in low income settings are warranted.

Multi-nutrient and prebiotic fortification of breast milk shows significant short-term effects but, apart from limited evidence of a lack of effect on psychomotor development at 18 months, evidence on longer-term effect is lacking. Fortifying breast milk with fat alone was not shown to confer any benefit but these results were from a small study and, therefore, the evidence was limited. Fortifying feeds with fat

Confidential: For Review Only

may improve longer term outcomes, given the theoretical benefit of fats on cognition and neurodevelopment.[32] Larger trials in LMIC settings, designed and powered to measure the long-term effects of breast milk fortifiers on growth and development, the incidence of adverse events as well as cost effectiveness are desirable. However, research results may not be applicable until multi-nutrient fortifiers become readily available and affordable in LMICs. A pragmatic alternative would be to test the effects of term formula milk powder for supplementation of calories and macronutrients with due attention to possible adverse effects.

Conclusion

Evidence that informs basic feeding practices of VLBW/very preterm infants in low-resource settings in sSA is poor. There is an urgent need for trials that evaluate pragmatic, inexpensive, low technology and sustainable feeding interventions. Follow-up is needed to include longer-term growth and neurodevelopmental outcomes.

Acknowledgements:

Dr. Symon Kariuki and Kenya Medical Research Institute (KEMRI) librarian Alex Maina assisted with the search and obtaining full texts of reviews. This paper is published with the permission of the Director of the KEMRI-Wellcome Trust Research Programme (Kilifi Centre).

Declaration of interest: No conflicts of interest to declare.

Sources of support/funding: This overview is supported by an MRC Confidence in Global Nutrition and Health Research Initiative grant: Improving the survival, growth and development of low birth weight newborns through better nutrition (Reference:

Confidential: For Review Only

MC_PC_MR/R019789/1). The funders had no role in the design or execution of the study, or preparation of the manuscript.

Contributors: AA conceived the study, undertook the literature search, selected reviews, extracted data and wrote the first draft of the manuscript. AT conceived the study, undertook the literature search, selected reviews, checked extracted data and wrote the first draft of the manuscript. SJA conceived the study, arbitrated on selection of reviews, revised the manuscript. IS advised on design of the study, revised the manuscript. NDE advised on design of the study, revised the manuscript All authors read and approved the final manuscript.

Exclusive licence: I, the Submitting Author has the right to grant and does grant on behalf of all authors of the Work (as defined in the below author licence), an exclusive licence and/or a non-exclusive licence for contributions from authors who are: i) UK Crown employees; ii) where BMJ has agreed a CC-BY licence shall apply, and/or iii) in accordance with the terms applicable for US Federal Government officers or employees acting as part of their official duties; on a worldwide, perpetual, irrevocable, royalty-free basis to BMJ Publishing Group Ltd ("BMJ") its licensees and where the relevant Journal is co-owned by BMJ to the co-owners of the Journal, to publish the Work in BMJ Paediatrics Open and any other BMJ products and to exploit all rights, as set out in our licence.

The Submitting Author accepts and understands that any supply made under these terms is made by BMJ to the Submitting Author unless you are acting as an employee on behalf of your employer or a postgraduate student of an affiliated institution which is paying any applicable article publishing charge ("APC") for Open

Confidential: For Review Only

Access articles. Where the Submitting Author wishes to make the Work available on an Open Access basis (and intends to pay the relevant APC), the terms of reuse of such Open Access shall be governed by a Creative Commons licence – details of these licences and which Creative Commons licence will apply to this Work are set out in our licence referred to above.

Confidential: For Review Only

References

1. Chawanpaiboon S, Vogel JP, Moller AB, et al. Global, regional, and national estimates of levels of preterm birth in 2014: a systematic review and modelling analysis. *Lancet Glob Health* 2019;7(1):e37-e46. doi: 10.1016/S2214-109X(18)30451-0 [published Online First: 2018/11/06]

2. Grandi C, Gonzalez A, Zubizarreta J, et al. Perinatal factors associated with neonatal mortality in very low birth weight infants: a multicenter study. *Arch Argent Pediatr* 2016;114(5):426-33. doi: 10.5546/aap.2016.eng.426

10.5546/aap.2016.426 [published Online First: 2016/09/09]

3. Latal-Hajnal B, von Siebenthal K, Kovari H, et al. Postnatal growth in VLBW infants: significant association with neurodevelopmental outcome. *J Pediatr* 2003;143(2):163-70. doi: 10.1067/S0022-3476(03)00243-9 [published Online First: 2003/09/13]

4. Abitbol CL, Moxey-Mims M. Chronic kidney disease: Low birth weight and the global burden of kidney disease. *Nat Rev Nephrol* 2016;12(4):199-200. doi: 10.1038/nrneph.2016.19 [published Online First: 2016/02/24]

5. O'Leary M, Edmond K, Floyd S, et al. A cohort study of low birth weight and health outcomes in the first year of life, Ghana. *Bull World Health Organ* 2017;95(8):574-83. doi: 10.2471/BLT.16.180273 [published Online First: 2017/08/15]

6. Cacho NT, Lawrence RM. Innate Immunity and Breast Milk. *Front Immunol* 2017;8:584. doi: 10.3389/fimmu.2017.00584 [published Online First: 2017/06/15]

Confidential: For Review Only

7. Lewandowski AJ, Lamata P, Francis JM, et al. Breast Milk Consumption in Preterm Neonates and Cardiac Shape in Adulthood. *Pediatrics* 2016;138(1) doi: 10.1542/peds.2016-0050 [published Online First: 2016/06/16]

8. Lee AC, Panchal P, Folger L, et al. Diagnostic Accuracy of Neonatal Assessment for Gestational Age Determination: A Systematic Review. *Pediatrics* 2017;140(6) doi: 10.1542/peds.2017-1423 [published Online First: 2017/11/19]

9. Organization WH. Guidelines on optimal feeding of low birth weight babies in low and middle income countries. Geneva: WHO 2011.

10. Mosley WH, Chen LC. An analytical framework for the study of child survival in developing countries. 1984. *Bull World Health Organ* 2003;81(2):140-5. [published Online First: 2003/05/22]

11. Nyamtema AS, Urassa DP, van Roosmalen J. Maternal health interventions in resource limited countries: a systematic review of packages, impacts and factors for change. *BMC Pregnancy Childbirth* 2011;11:30. doi: 10.1186/1471-2393-11-30 [published Online First: 2011/04/19]

12. Bradley S, McAuliffe E. Mid-level providers in emergency obstetric and newborn health care: factors affecting their performance and retention within the Malawian health system. *Hum Resour Health* 2009;7:14. doi: 10.1186/1478-4491-7-14 [published Online First: 2009/02/21]

13. Quigley M, Embleton ND, McGuire W. Formula versus donor breast milk for feeding preterm or low birth weight infants. *Cochrane Database Syst Rev* 2018;6:CD002971. doi: 10.1002/14651858.CD002971.pub4 [published Online First: 2018/06/22]

Confidential: For Review Only

14. Watson J, McGuire W. Transpyloric versus gastric tube feeding for preterm infants. *Cochrane Database Syst Rev* 2013(2):CD003487. doi: 10.1002/14651858.CD003487.pub3 [published Online First: 2013/03/02]

15. Tan-Dy CR, Ohlsson A. Lactase treated feeds to promote growth and feeding tolerance in preterm infants. *Cochrane Database Syst Rev* 2013(3):CD004591. doi: 10.1002/14651858.CD004591.pub3 [published Online First: 2013/04/02]

16. Klenoff-Brumberg HL, Genen LH. High versus low medium chain triglyceride content of formula for promoting short term growth of preterm infants. *Cochrane Database Syst Rev* 2003(1):CD002777. doi: 10.1002/14651858.CD002777 [published Online First: 2003/01/22]

17. Verner A, Craig S, McGuire W. Effect of taurine supplementation on growth and development in preterm or low birth weight infants. *Cochrane Database Syst Rev* 2007(4):CD006072. doi: 10.1002/14651858.CD006072.pub2 [published Online First: 2007/10/19]

18. Shea BJ, Reeves BC, Wells G, et al. AMSTAR 2: a critical appraisal tool for systematic reviews that include randomised or non-randomised studies of healthcare interventions, or both. *BMJ* 2017;358:j4008. doi: 10.1136/bmj.j4008 [published Online First: 2017/09/25]

19. Cochrane Handbook for Systematic Reviews of Interventions Version 5.1.0 [updated March 2011]: The Cochrane Collaboration 2011.

20. Brown JV, Embleton ND, Harding JE, et al. Multi-nutrient fortification of human milk for preterm infants. *Cochrane Database Syst Rev* 2016(5):CD000343. doi: 10.1002/14651858.CD000343.pub3 [published Online First: 2016/05/09]

21. Premji SS, Chessell L. Continuous nasogastric milk feeding versus intermittent bolus milk feeding for premature infants less than 1500 grams. *Cochrane*

Confidential: For Review Only

Database Syst Rev 2011(11):CD001819. doi: 10.1002/14651858.CD001819.pub2 [published Online First: 2011/11/11]

22. Oddie SJ, Young L, McGuire W. Slow advancement of enteral feed volumes to prevent necrotising enterocolitis in very low birth weight infants. *Cochrane Database Syst Rev* 2017;8:CD001241. doi: 10.1002/14651858.CD001241.pub7 [published Online First: 2017/08/31]
23. Alshaikh B, Dharel D, Yusuf K, et al. Early total enteral feeding in stable preterm infants: a systematic review and meta-analysis. *J Matern Fetal Neonatal Med* 2019:1-8. doi: 10.1080/14767058.2019.1637848 [published Online First: 2019/06/30]
24. Abiramalatha T, Thomas N, Gupta V, et al. High versus standard volume enteral feeds to promote growth in preterm or low birth weight infants. *Cochrane Database Syst Rev* 2017;9:CD012413. doi: 10.1002/14651858.CD012413.pub2 [published Online First: 2017/09/13]
25. Amissah EA, Brown J, Harding JE. Fat supplementation of human milk for promoting growth in preterm infants. *Cochrane Database Syst Rev* 2018;6:CD000341. doi: 10.1002/14651858.CD000341.pub2 [published Online First: 2018/06/21]
26. Amissah EA, Brown J, Harding JE. Carbohydrate supplementation of human milk to promote growth in preterm infants. *Cochrane Database Syst Rev* 2018;8:CD000280. doi: 10.1002/14651858.CD000280.pub2 [published Online First: 2018/08/24]
27. Watson J, McGuire W. Nasal versus oral route for placing feeding tubes in preterm or low birth weight infants. *Cochrane Database Syst Rev* 2013(2):CD003952. doi: 10.1002/14651858.CD003952.pub3 [published Online First: 2013/03/02]

Confidential: For Review Only

28. Kimani-Murage EW, Wanjohi MN, Kamande EW, et al. Perceptions on donated human milk and human milk banking in Nairobi, Kenya. *Matern Child Nutr* 2019;15(4):e12842. doi: 10.1111/mcn.12842 [published Online First: 2019/05/18]

29. Magowan S, Burgoine K, Ogara C, et al. Exploring the barriers and facilitators to the acceptability of donor human milk in eastern Uganda - a qualitative study. *Int Breastfeed J* 2020;15(1):28. doi: 10.1186/s13006-020-00272-1 [published Online First: 2020/04/19]

30. Morgan J, Young L, McGuire W. Delayed introduction of progressive enteral feeds to prevent necrotising enterocolitis in very low birth weight infants. *Cochrane Database Syst Rev* 2014(12):CD001970. doi: 10.1002/14651858.CD001970.pub5 [published Online First: 2014/12/02]

31. Mulvihill SJ, Stone MM, Debas HT, et al. The role of amniotic fluid in fetal nutrition. *J Pediatr Surg* 1985;20(6):668-72. doi: 10.1016/s0022-3468(85)80021-x [published Online First: 1985/12/01]

32. Delplanque B, Gibson R, Koletzko B, et al. Lipid Quality in Infant Nutrition: Current Knowledge and Future Opportunities. *J Pediatr Gastroenterol Nutr* 2015;61(1):8-17. doi: 10.1097/MPG.0000000000000818 [published Online First: 2015/04/18]

Confidential: For Review Only

Figure 1 Flow diagram of selection of reviews

Confidential: For Review Only

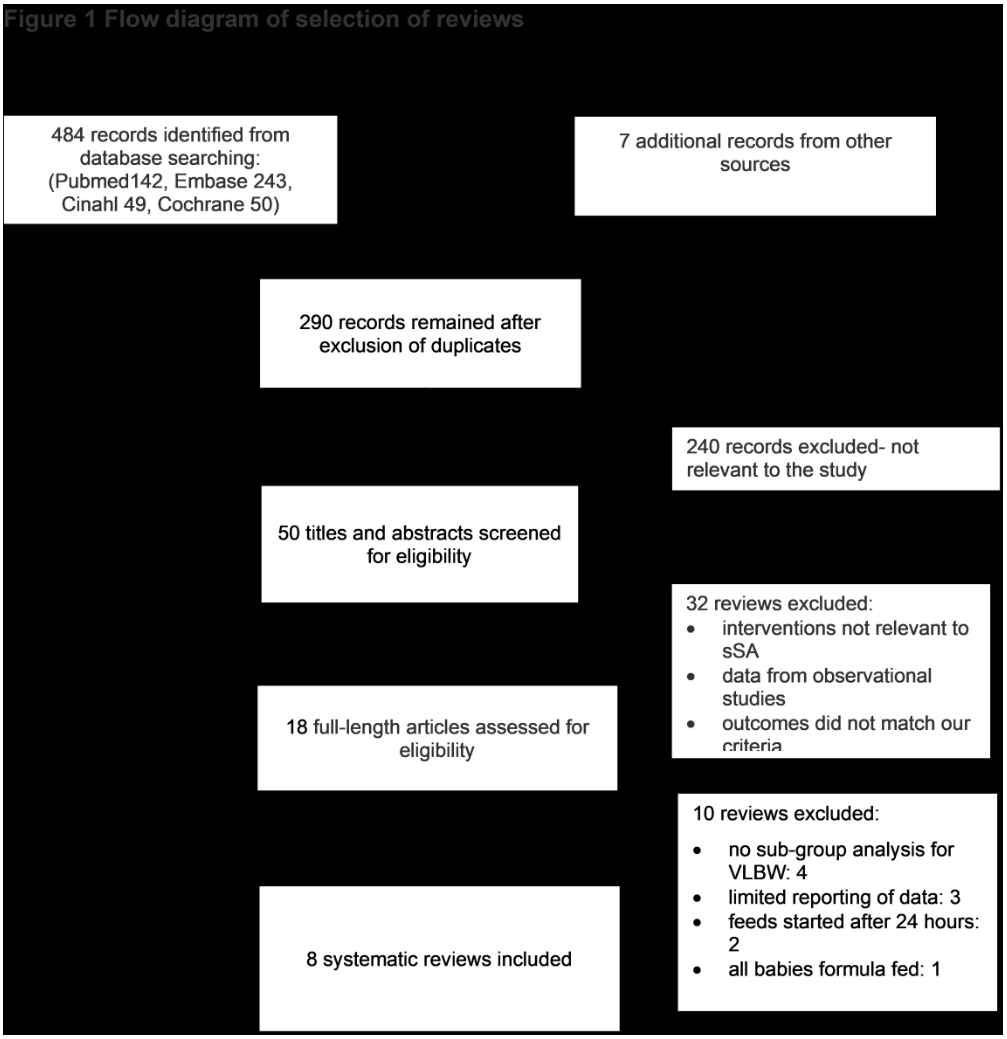

Figure 1 Flow diagram of selection of reviews

Characteristics of Excluded Reviews

Review	Reason for exclusion
Flint 2016[1]	Included term babies. No sub-group analysis for VLBW infants
Morgan 2013[2]	Trophic feeds did not start before 24 hours
Morgan 2014[3]	Enteral feeds did not start before 24 hours
Richards 2014[4]	No RCTs on VLBW infants
Dawson 2012[5]	No sub-group analysis for VLBW infants
Moon 2016[6]	All babies formula fed
Kwok 2019[7]	Limited reporting of growth data
Civardi 2015[8]	Meeting abstract- limited reporting of data
Amissah 2018[9]	No sub-group analysis for VLBW infants
De Pipaon 2019[10]	Meeting abstract – limited reporting of data

References

1. Flint A, New K, Davies MW. Cup feeding versus other forms of supplemental enteral feeding for newborn infants unable to fully breastfeed. *Cochrane Database Syst Rev* 2016(8):CD005092. doi: 10.1002/14651858.CD005092.pub3 [published Online First: 2016/09/01]

2. Morgan J, Bombell S, McGuire W. Early trophic feeding versus enteral fasting for very preterm or very low birth weight infants. *Cochrane Database Syst Rev* 2013(3):CD000504. doi: 10.1002/14651858.CD000504.pub4 [published Online First: 2013/04/02]

Confidential: For Review Only

3. Morgan J, Young L, McGuire W. Delayed introduction of progressive enteral feeds to prevent necrotising enterocolitis in very low birth weight infants. *Cochrane Database Syst Rev* 2014(12):CD001970. doi: 10.1002/14651858.CD001970.pub5 [published Online First: 2014/12/02]

4. Richards R, Foster JP, Psaila K. Continuous versus bolus intragastric tube feeding for preterm and low birth weight infants with gastro-oesophageal reflux disease. *Cochrane Database Syst Rev* 2014(7):CD009719. doi: 10.1002/14651858.CD009719.pub2 [published Online First: 2014/07/18]

5. Dawson JA, Summan R, Badawi N, et al. Push versus gravity for intermittent bolus gavage tube feeding of premature and low birth weight infants. *Cochrane Database Syst Rev* 2012;11:CD005249. doi: 10.1002/14651858.CD005249.pub2 [published Online First: 2012/11/16]

6. Moon K, Rao SC, Schulzke SM, et al. Longchain polyunsaturated fatty acid supplementation in preterm infants. *Cochrane Database Syst Rev* 2016;12:CD000375. doi: 10.1002/14651858.CD000375.pub5 [published Online First: 2016/12/21]

7. Kwok TC, Dorling J, Gale C. Early enteral feeding in preterm infants. *Semin Perinatol* 2019;43(7):151159. doi: 10.1053/j.semperi.2019.06.007 [published Online First: 2019/08/25]

8. Civardi E. Trophic feeding for very preterm or very low birth weight infants. *Italian Journal of Pediatrics* 2015;41(Suppl 1):A3.

9. Amissah EA, Brown J, Harding JE. Protein supplementation of human milk for promoting growth in preterm infants. *Cochrane Database Syst Rev* 2018;6:CD000433. doi: 10.1002/14651858.CD000433.pub2 [published Online First: 2018/06/23]

10. De Pipaon M.S. LMR, Couce M.L., Sanz-Valero J. Effect of long-chain polyunsaturated fatty acid supplementation of preterm infants on infection. A systematic review and meta-analysis. *J Pediatr Gastroenterol Nutr* 2019;68(Supplement 1):1005-06.

Confidential: For Review Only
